# Parallel signatures of *Mycobacterium tuberculosis* and human Y-chromosome phylogeography support the Two Layer model of East Asian population history

**DOI:** 10.1038/s42003-023-05388-8

**Published:** 2023-10-13

**Authors:** Matthew Silcocks, Sarah J. Dunstan

**Affiliations:** Department of Infectious Diseases, University of Melbourne at the Peter Doherty Institute for Infection and Immunity, Parkville, VIC Australia

**Keywords:** Coevolution, Population genetics, Phylogenetics, Archaeology, Bacterial genetics

## Abstract

The Two Layer hypothesis is fast becoming the favoured narrative describing East Asian population history. Under this model, hunter-gatherer groups who initially peopled East Asia via a route south of the Himalayas were assimilated by agriculturalist migrants who arrived via a northern route across Eurasia. A lack of ancient samples from tropical East Asia limits the resolution of this model. We consider insight afforded by patterns of variation within the human pathogen *Mycobacterium tuberculosis (Mtb)* by analysing its phylogeographic signatures jointly with the human Y-chromosome. We demonstrate the Y-chromosome lineages enriched in the traditionally hunter-gatherer groups associated with East Asia’s first layer of peopling to display deep roots, low long-term effective population size, and diversity patterns consistent with a southern entry route. These characteristics mirror those of the evolutionarily ancient *Mtb* lineage 1. The remaining East Asian Y-chromosome lineage is almost entirely absent from traditionally hunter-gatherer groups and displays spatial and temporal characteristics which are incompatible with a southern entry route, and which link it to the development of agriculture in modern-day China. These characteristics mirror those of the evolutionarily modern *Mtb* lineage 2. This model paves the way for novel host-pathogen coevolutionary research hypotheses in East Asia.

## Abstract (web summary)

The phylogeographic patterns of *Mycobacterium tuberculosis* lineages in East Asia mirror those of human Y-chromosome haplogroups, supporting the Two Layer hypothesis, and a Paleolithic emergence scenario for the *Mycobacterium tuberculosis* complex.

## Introduction

With contributions from the fields of anthropometry and the study of ancient and contemporary DNA, a new paradigm has emerged in our understanding of East Asian population history^1–5^. This newly emerged consensus holds that East Asia was peopled in two main population movements or layers. The first layer consisted of hunter-gatherer groups with darkly pigmented skin, who arrived in East Asia via a dispersal route south of the Himalaya mountain range. These populations were later displaced and partially assimilated by agriculturalist groups with cold adapted physical features, who were likely to have reached East Asia via a route north of the Himalayas^2^.

Interestingly, the Two Layer model, and the closely related Dual Structure model of Japanese population history were originally rooted in the field of physical anthropology and received support from genetic studies only recently. The Two Layer model was initially proposed to explain the replacement of Hoabinhian hunter-gatherers exhibiting Australo-Melanesian features with agriculturalist groups exhibiting Northeast Asian features in the Southeast Asian archaeological record^2,6–10^. Similarly, the Dual Structure model describes the displacement and integration of hunter-gatherer Jomon groups by agriculturalist migrants across the Japanese archipelago. Under this theory, which draws from skeletal, odontal and craniometric data, Jomon-related ancestry was argued to persist mainly in the Indigenous Ainu and Ryukyuan groups from the northern and southern extremes of the island chain^11–20^. Genetic studies garnered support for both the Two Layer and Dual Structure models, by demonstrating the strong degree of shared genetic drift between ancient Jomon and modern Ainu genomes^21^, and between ancient Jomon and Hoabinhian genomes^3^.

Current studies now describe East Asian populations, at a basal level, to be modelled as mixtures of first and second layer ancestry, with the Indigenous Onge and the ancient Tianyuan genome from Neolithic northern China acting as surrogates for each respective layer^1^. Under these models, the Onge are argued to be amongst a number of modern-day human populations who represent largely unadmixed descendants of the first layer of peopling, including Papuans, the Ainu of Japan, and hunter-gatherers from the Philippines and Malaysia^1,2,4^.

While analyses of ancient DNA have afforded great insight into the Two Layer model, they are limited by the low availability of this form of data from the region. An underutilised means of deciphering aspects of human demography is by analysing patterns of genetic variation amongst human symbiotic species, including commensals and pathogens^22–24^.

In this study, we consider the insight afforded into the Two Layer model by analysing genetic variation within the genomes of the human obligate pathogen *Mtb*. We complement these inferences by investigating patterns of human genetic variation, inferred from Y-chromosome sequencing and genotype data. We pair these two marker systems, as they both possess ample phylogenetic signal, and are both inherited clonally, thus permitting the use of an identical suite of phylogenetic and phylogeographic tools in their analysis. The joint analysis of variation in these marker systems may reveal parallel phylodynamic and geographic signatures, which may be relevant to our understanding of the Two Layer model, and may furnish us with additional insights. We are also motivated to complete this investigation, as existing models describing the distribution of both Y-chromosome and *Mtb* genetic variation are largely incompatible with the Two Layer hypothesis.

Y-Chromosome data, while having played a seminal role in illuminating our African origins^25,26^, and describing male mediated demographic processes^27,28^, has not been robustly reconciled with the Two Layer model. Current models of East Asian Y-chromosome variation generally argue a single southern route origin of the four haplogroups which make up 90–95% of male lineages [C, D, and the NO clade (comprising haplogroups N and O)^29–32^], followed by subsequent northwards expansion^30,31,33^. These theories therefore don’t account for the proposed northern dispersal route associated with the second layer of peopling, or the implied genetic affinities shared by the present-day representatives of the first layer.

Similarly, genetic variation in the *Mtb* pathogen has also been well characterised in an East Asian context but has not been assessed in light of the Two Layer model. The East Asian *Mtb* landscape is dominated by isolates from the ‘evolutionarily ancient’ lineage 1, found at highest frequency in southern regions, and the ‘evolutionarily modern’ lineage 2, which display enhanced virulence and transmissibility^34–36^, and which predominates in northerly regions^37^. Under models proposing a Paleolithic^38,39^, as opposed to an argued Neolithic^34,37,40,41^ origin of *Mtb*, lineage 2 arrived in East Asia via a southern route, before expanding northwards, and radiating southwards again with the movement of the Han people^39^. This model doesn’t reconcile all aspects of the Two Layer hypothesis, including the proposed northern entry route of the second layer, nor the demographic processes and migrations which gave rise to the distribution of lineage 1.

Here we conduct a joint analysis of patterns of *Mtb* and Y-chromosome variation to propose a coevolutionary scenario compatible with the Two Layer model. In addition to illuminating a key chapter of human history, it will provide background context relevant to our understanding of the long-term interaction of humans and one of the deadliest diseases of all time.

## Results

### Y-chromosome variation and the Two Layer model

To explore East Asian patrilineal diversity we collated a large dataset of Y-chromosome haplogroup population frequencies. Studies were selected so as to unambiguously assign haplogroups to a fine degree of phylogenetic resolution^42^, and to cover a wide geographical extent (Supplementary Note 1; Supplementary Fig. 1).

Consistent with the results of previous studies^31,33^, we found haplogroups C, D and NO to account for around 95% of East Asian male lineages (Supplementary Note 1; Supplementary Figs. 2–7). When incorporating populations from Island Southeast Asia and Oceania, the K2b1 lineage, and three minor sister groups, K2c, K2d and P*, which are indistinguishable in most genotyping platforms, emerge as the fourth predominant lineage^43^.

We observed low frequencies of Q, R, F* and H at the western fringes of East Asia (Supplementary Note 1; Supplementary Figs. 8–11). We did not consider these lineages further, as prior studies have inferred a Central/North Eurasian origin for the former two^31^ (Supplementary Note 1; Supplementary Figs. 8, 9) and a South Asian origin for the latter pair^44,45^ (Supplementary Note 1; Supplementary Figs. 10, 11).

**Fig. 1:**
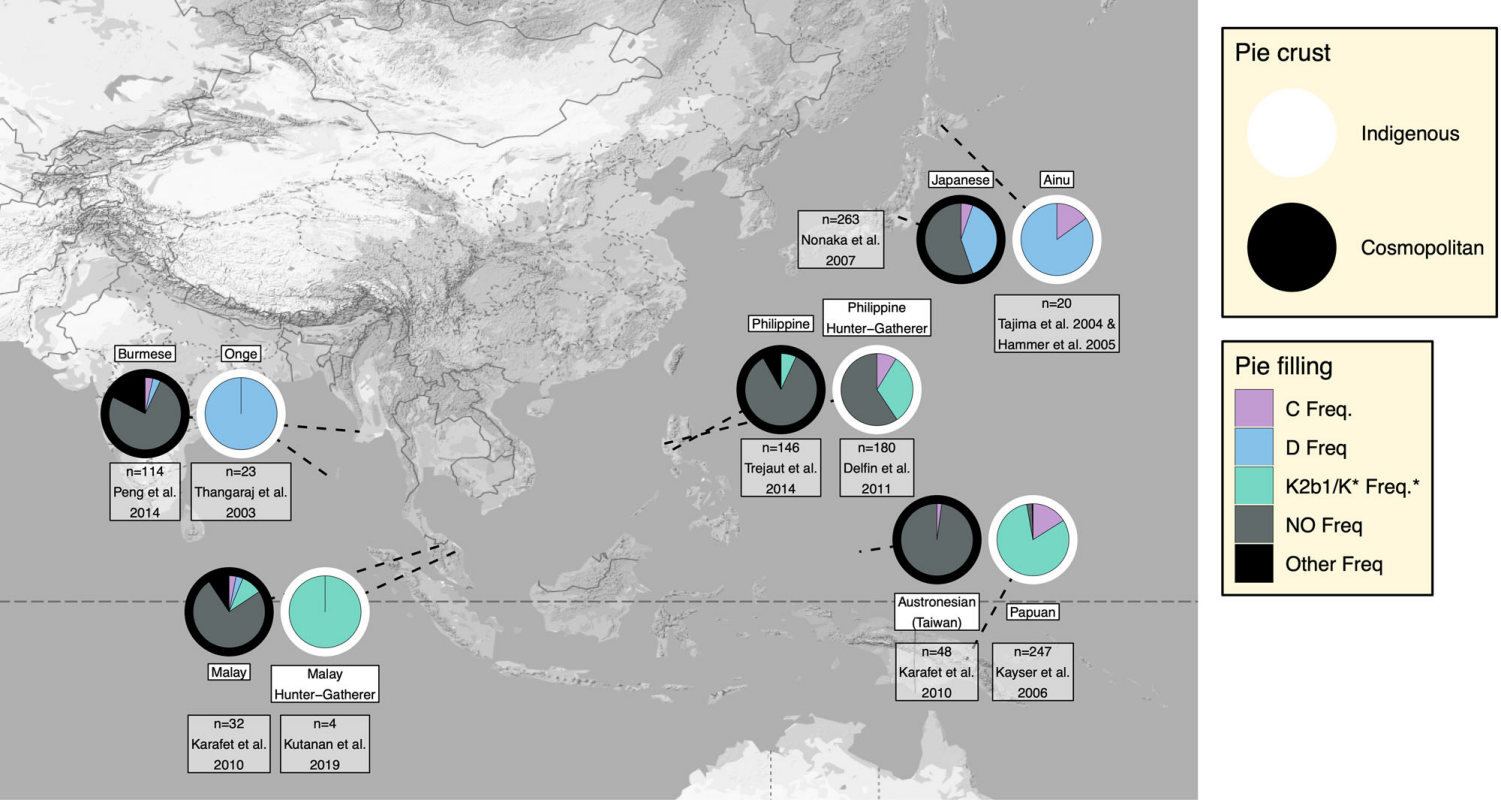
Y-chromosome haplogroup proportions in traditionally hunter-gatherer first layer populations of East Asia, and nearby cosmopolitan groups (Designated with white and black pie borders respectively). The four Y-chromosome haplogroups which predominate in East Asian populations (C, D, K2b1/K* and NO) are designated with pink, blue, green and grey pie fillings respectively, with all other haplogroups designated black. *Note that in some datasets from which samples were drawn, the markers required to designate a K* haplotype as K2b1 were not typed, meaning that a small proportion of haplotypes in this category may belong to the rare haplogroups K2c, K2d and P* which have been shown to be entirely restricted to Island Southeast Asia^43^. Map was sourced from Google Maps using the using the ‘get_googlemap’ function of ggmap^129^.

We also considered the frequencies of haplogroups summarised in previous studies involving traditionally hunter-gatherer groups associated with the first layer peopling of East Asia^2–4,9^ (Supplementary Note 1) and paired each with a geographically proximate non-Indigenous population. Doing so revealed a pattern of unity amongst the Indigenous populations, with the enrichment of lineages C, D and K2b1 when compared to each non-Indigenous group, which overwhelmingly carried lineages from the NO clade (Fig. 1).

Next, we explored the evolutionary dynamics of these Y-chromosome lineages, which can be inferred backwards across time using a phylogenetic toolkit. We inferred a phylogeny from Y-chromosome whole genome sequences from the Human Genome Diversity Panel (HGDP)^46^, which covers a wide and balanced selection of East Asian populations. To infer trajectories of effective population size for each of our four haplogroups we applied the Bayesian skyline technique, which does so from the distribution and spacing of coalescence events in that phylogeny^47,48^. Typically, this technique is applied to sequences sampled from a single homogenous population, however it is often applied to individual haplogroups when one has prior reason to associate a specific lineage with a particular population or movement of people^49–54^.

**Fig. 2:**
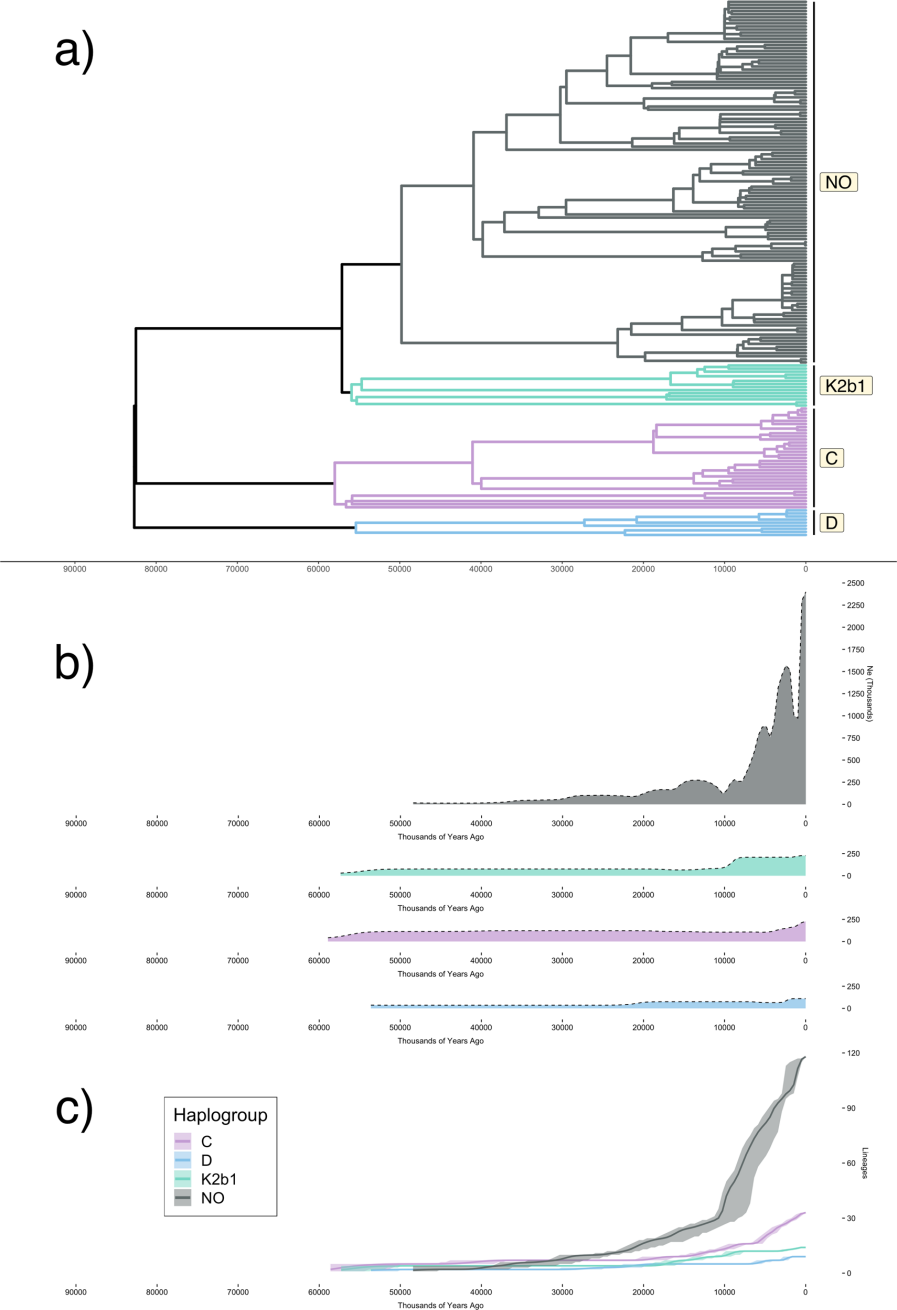
Dynamics of the predominant East Asian Y-chromosome lineages inferred from the HGDP. **a** Phylogeny of Y-chromosome haplogroups NO, K2b1, C and D. **b** Bayesian skyline plots estimating effective population size through time for each of these four haplogroups. The colour scheme used to designate each of these lineages is the same across all figure panels, and that used in Fig. 1. **c** Plot of lineages through time for each of the four Y-chromosome haplogroups, with shading indicating 95% highest posterior density (HPD) estimates across all iterations sampled by BEAST.

We see a strong contrast between the inferred historic populations sizes of the NO clade, and the three lineages which predominate in traditionally hunter-gatherer groups: C, D and K2b1 (Fig. 2). Lineages C, D and K2b1 are deeply rooted (Fig. 2a), with coalescence times exceeding 50Kya, and each being inferred to have sustained low population sizes until the present (Fig. 2b).

The NO clade, which diversified more recently, experiences an initial population size increase around 15Kya, and begins a phase of exponential growth around 9Kya, expanding by a factor of almost 3 within the next 3000 years (Fig. 2b; from ~278,000 at 9Kya to ~776,000 at 6Kya). It is notable that the timeframe for this latter episode of growth coincides with the development of agriculture, which is inferred from archaeological data to have begun around 7000–6000BCE in Northern China^55,56^.

Importantly, we show that the contrasting temporal trends of the two sets of Y-chromosome lineages are consistent under different parameter choices for the skyline technique, when considering N and O lineages separately, and when considering the simpler ‘lineages through time’ trajectory for each haplogroup (Fig. 2c; Supplementary Note 2; Supplementary Figs. 14–16).

**Fig. 3:**
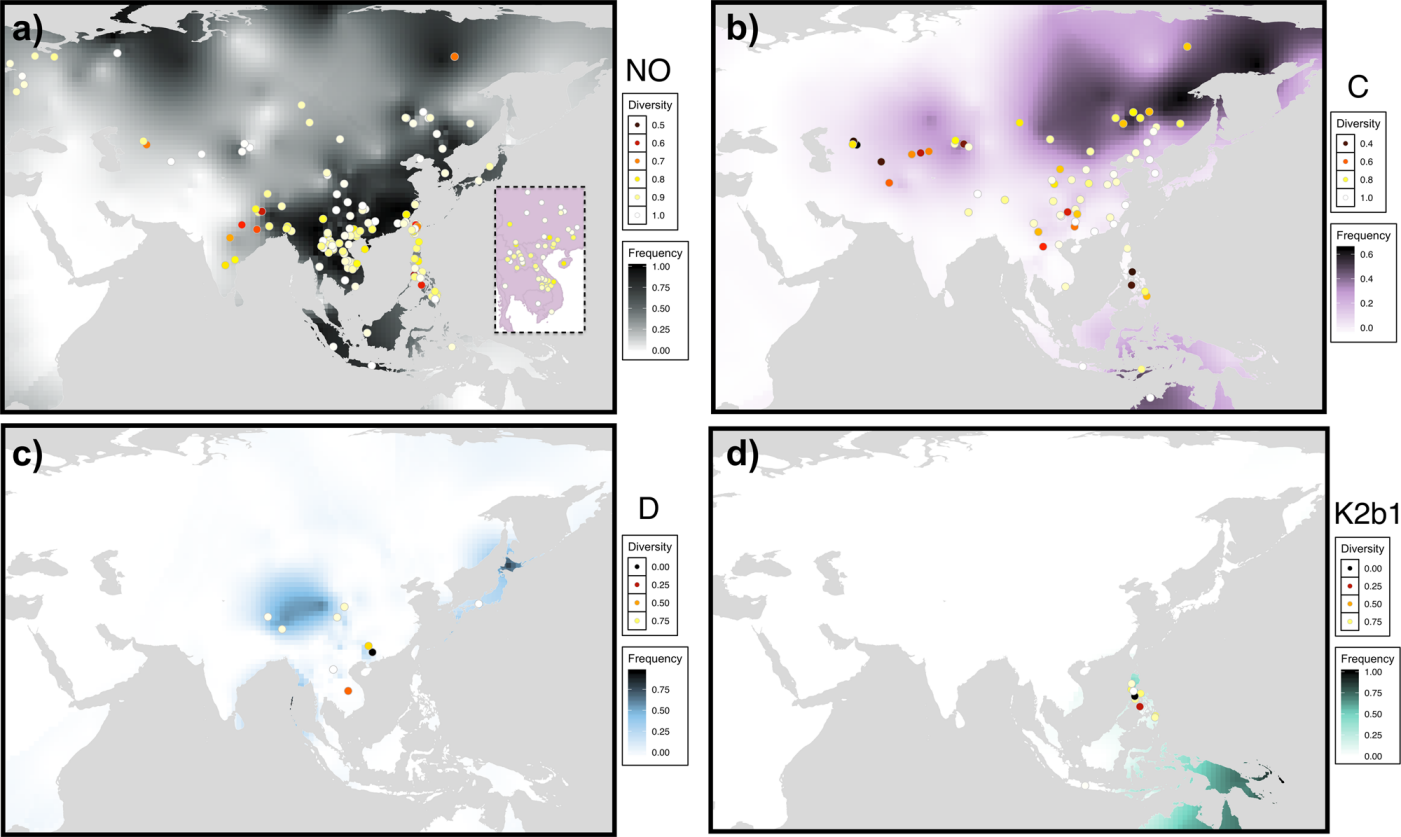
Spatial frequency distributions of the four major East Asian Y-chromosome lineages (surface colour gradient) with points showing haplogroup diversity estimated from 6 STRs. **a** Haplogroup NO. **b** Haplogroup C. **c** Haplogroup D. **d** Haplogroup K2b1. Frequency and diversity colour scales differ between plots. Note that for the interpolation of haplogroup K2b1, only lineages which could be unambiguously assigned to this clade, by possessing derived alleles at markers for its sub-lineages M or S, were counted towards the frequency of this haplogroup. Several Indonesian populations possessed low frequencies of K-M526*. These lineages have been shown by Karafet et al. (2015)^43^ to mainly belong to K2b1, so the frequency of this lineage is likely to be slightly underestimated in these few Island Southeast Asian populations. Maps were obtained using the ggmap^129^ package of R.

We next used the spatial frequency interpolation technique ‘kriging’ to visualise the distributions of the four predominant East Asian lineages, and assess these in light of the Two Layer model (Fig. 3a–d). The maps of population haplogroup frequencies were supplemented with measures of haplogroup diversity, generated from a dataset of over 5,000 STR profiles (Fig. 3; points). These techniques have a long history of use in human population genetics^29–32,57^ and allow researchers to infer likely origin points of haplogroups from regions of high diversity^58–61^.

From this analysis we observe that haplogroup C is found across a wide expanse of East Asia, Island Southeast Asia and Oceania, and at particularly high frequencies in inland and Northern Eurasia (Fig. 3b). We observe that estimates of haplogroup C diversity in inland and northern Eurasia are extremely low relative to modern-day China. Haplogroup D is found at highest frequencies across Tibet, Japan and the Andaman Islands, but also has a patchy distribution across mainland Southeast Asia (Fig. 3c). Haplogroup K2b1 is found exclusively in Papua New Guinea (PNG), Australia, the Philippines and Eastern Indonesia (Fig. 3d). Haplogroup NO is widely distributed, and is the only haplogroup of the four found in North-Western Eurasia (Fig. 3a). The diversity of this lineage appears highest in modern-day China, and Southeast Asia, and drops off when moving east towards the Philippines, and more dramatically when moving west towards India.

Based on the spatial and temporal characteristics of lineages C, D and K2b1, and their enrichment in traditionally hunter-gatherer first layer populations, we infer them to have arrived via the initial southern coastal route migration into East Asia. The NO lineage, which is underrepresented or absent from these populations, and which displays signatures of exponential population growth and spatial characteristics incompatible with a southern entry route, we infer to have arrived via the second layer of peopling.

### *Mtb* genetic variation and the Two Layer model

In light of the above data, we now consider the distributions, frequencies, and phylogenetic characteristics of East Asian *Mtb* lineages. We visualised the spatial frequency of lineages 1 and 2 using data from a recent joint-analysis^62^ and supplemented these maps with estimates of lineage diversity inferred from spoligotypes from the SITVIT2 database^63^.

**Fig. 4:**
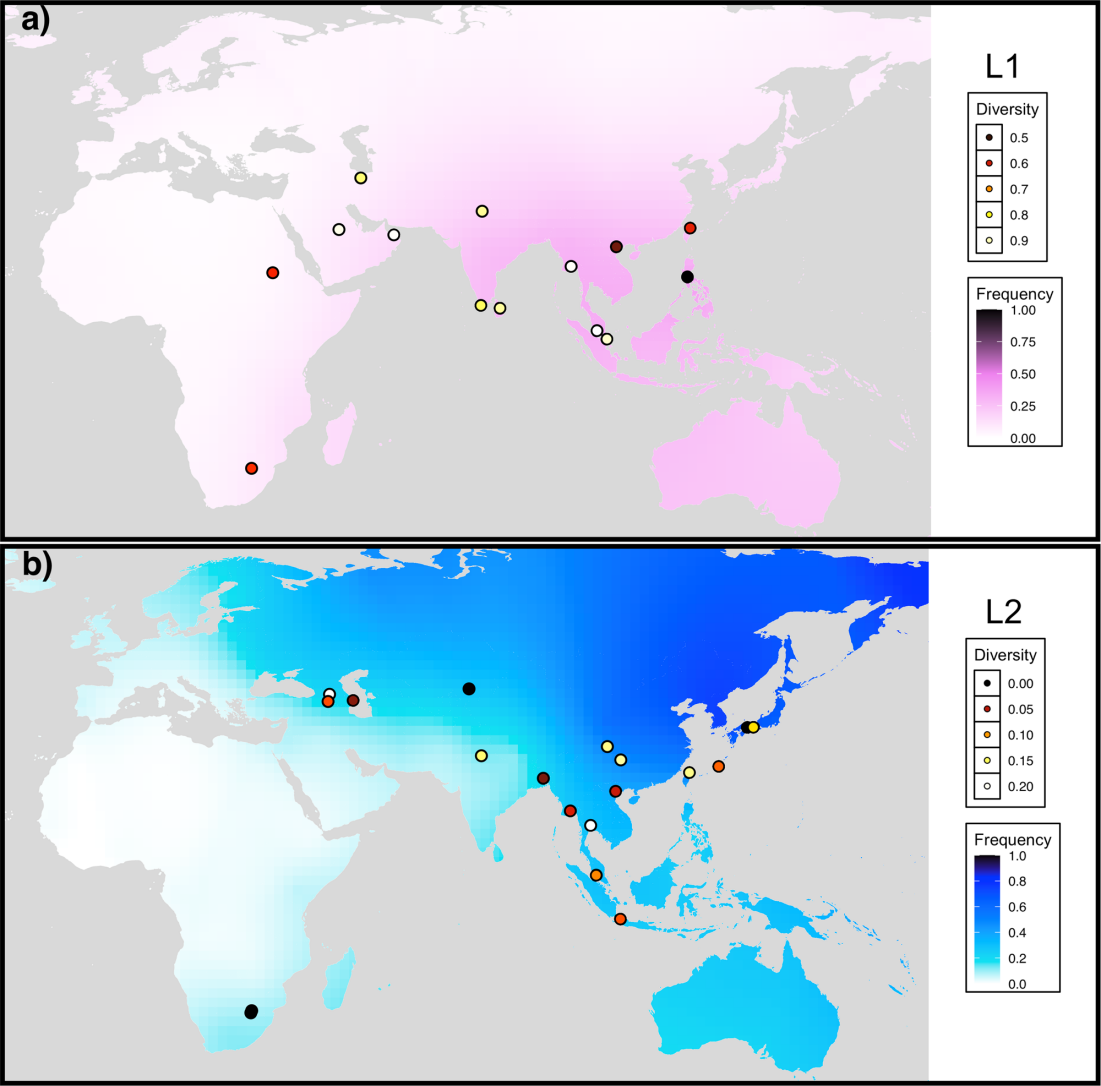
Spatial frequency distributions of *Mtb* lineages (surface colour gradient), with points showing lineage diversities estimated using spoligotype data from the SITVIT2 database. (**a**) lineage 1. (**b**) lineage 2. Note that the diversity colour scales differ between (**a**) and (**b**). Maps were obtained using the ggmap^129^ package of R.

We found lineage 1 to be distributed around the rim of the Indian Ocean, and to display uniformly high diversity values in the Asian countries which border it (Fig. 4a). Consistent with phylogenetic studies, we calculate low diversity values for lineage 1 in East African countries, as well as the Philippines, where the majority of *Mtb* lineages belong to the endemic ‘Manila’ lineage, which has a relatively young root^64^.

Lineage 2 is found at highest frequencies across Far East Asia, and extends west to Central Asia, as well as north to Russia (Fig. 4b). Lineage 2 isolates generally attain highest diversities in East Asian regions, including two sampling locations from China and one from Japan. The single highest L2 diversity value is reported for a sampling location in Bangkok, Thailand, however more southerly and westerly Southeast Asian locations consistently displayed lower diversity values. Much like Y-chromosome haplogroup NO, the frequency of lineage 2 drops when moving westwards across the Bay of Bengal into South Asia. Overall, levels of diversity, measured as the probability of selecting isolates with identical spoligotype patterns from a population, were far lower for lineage 2 than for lineage 1.

We propose these spatial frequency and diversity patterns suggest lineage 1 to show characteristics compatible with a southern route entry into East Asia, and lineage 2 to show opposing patterns, which are more consistent with a northern entry route, and more recent expansion. To support this association more formally, we assessed the correlation in frequency between putative first and second layer *Mtb* and Y-chromosome lineages across countries or major geographical regions in our two genotyping surveys. We found the frequencies of haplogroup NO and *Mtb* lineage 2 to show a strong positive correlation (Spearman’s rho = 0.65; *p* = 7.3 × 10^−4^), and the frequency of lineage 1 to be positively correlated with the summed frequencies of haplogroups C, D and K2b1 (Spearman’s rho = 0.44; *p* = 0.039; Supplementary Note 3; Supplementary Figs. 21 and 22).

Next, we inferred the phylogenetic characteristics of *Mtb* lineages 1 and 2 using a dataset of whole genome sequences, to observe features which may be shared with the Y-chromosome tree. We composed a dataset of isolates randomly sampled from available East Asian populations, which included Thailand, Myanmar, Cambodia, Vietnam, China and Japan. This sampling regime was designed to approximate the regime used for the HGDP, from which Y-chromosome dynamics were inferred. For the purposes of this analysis, we are examining qualitative similarities between the *Mtb* and Y-chromosome phylogenies, including relative divergence times and expansion trajectories of lineages putatively associated with each population movement. Due to inherent differences in the sampling designs of these studies, we did not expect exact congruence in the phylogenetic signatures observed.

As there is ongoing debate surrounding our ability to accurately estimate substitution rates in *Mtb*, and the stability of the instantaneous mutation rate of *Mtb* throughout time^65–67^, we chose not to calibrate our phylogeny according to a previously estimated rate. We chose instead to use the phylogenetic technique of ‘node calibration’, in which a tree is calibrated by assigning an age (or probability distribution over a range of ages) to a certain node, based off archaeological or biogeographic data, or signatures of co-divergence^68–71^. We chose this method in favour of the ‘tip calibration’ approach used by prior studies, which infer node ages and a substitution rate by incorporating heterochronous sequences into their phylogeny^72,73^. In addition to requiring adequate temporal structure^74,75^, tip dating is liable to the time dependent rate phenomenon^76,77^ (TDRP), which systematically underestimates the ages of deep nodes of a tree (see Discussion). Node calibration, while not subject to the TDRP, does, however, rely on a robust biogeographic or genetic model to justify the codivergence scenario proposed^72,73,78^ (see Discussion).

We used an approach analogous to Comas et al. (2013)^38^, who calibrate nodes of the *Mtb* phylogeny to match key divergence events in the human mitochondrial tree. As we infer *Mtb* lineage 1 to be the lineage carried by the first layer dispersal into East Asia, we assigned a prior distribution over its root age, such that it matches the average age of first layer Y-chromosome lineages C, D and K2b1 (56.5Ky, ±5Ky). Our inferred timepoint for the coalescence age of lineage 1 (56.5Kya) is therefore intermediate between the co-divergence scenarios proposed by Comas et al. (2013)^38^ (ranging from 6 to 100Kya), yet older than estimates derived from tip dating such as Menardo et al. (2021)^79^ (0.85Kya) and O’Neill et al. (2019)^37^ (2.38Kya; see Supplementary Note 5; Supplementary Table 1 for a comparison of models).

**Fig. 5:**
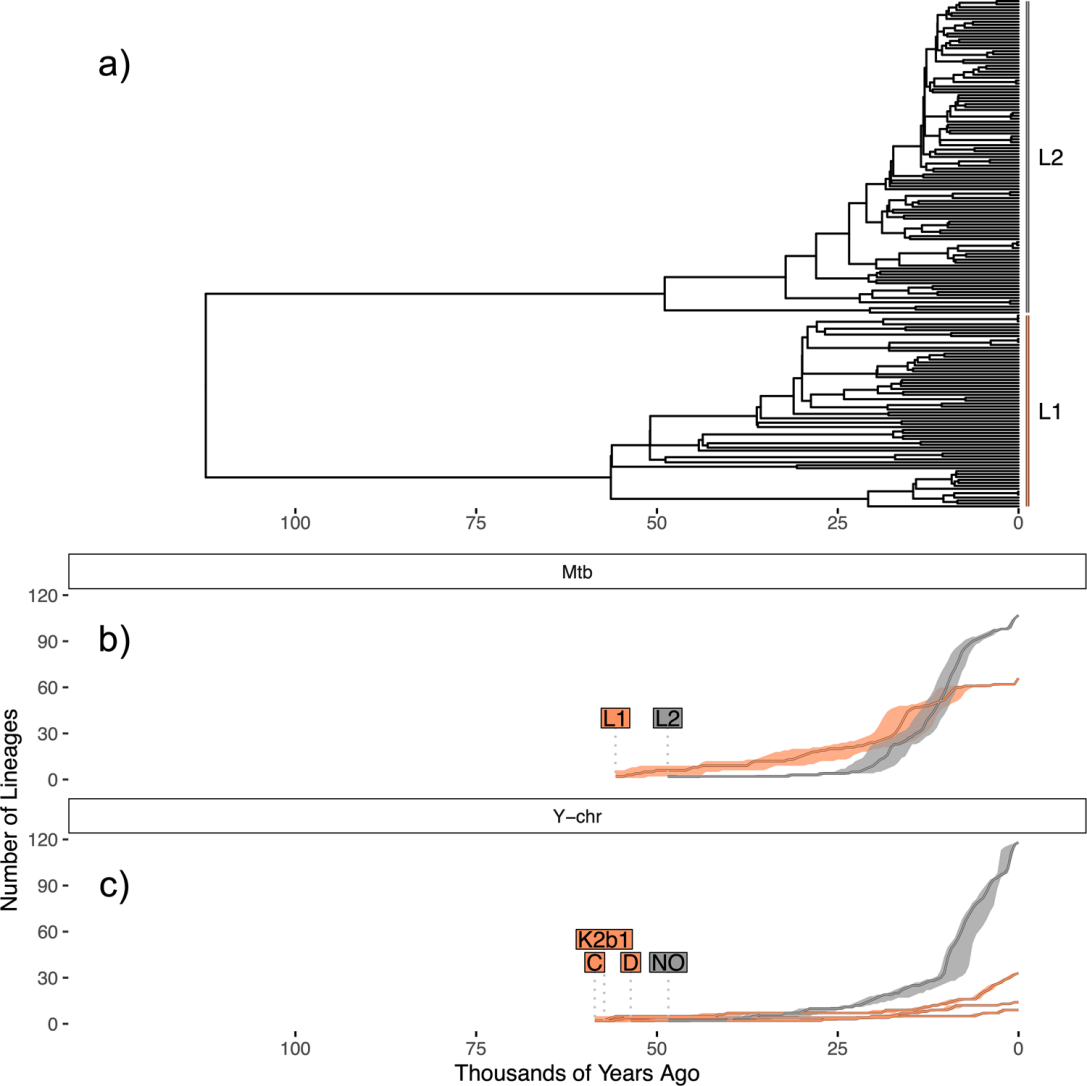
Dynamics of the predominant *Mtb* lineages in East Asia. **a** Phylogeny inferred from lineage 1 and 2 *Mtb* sequences from East Asian populations. **b** Lineage through time trajectories for L1 and L2 subtrees with 95% HPD intervals generated using BEAST. **c** Lineage through time trajectories for the four predominant East Asian Y-chromosome haplogroups shown in Fig. 2 with 95% HPD interval shading. The colour scheme used for (**b** and **c**) was devised so that orange corresponds to first layer *Mtb* and Y-chromosome lineages/ haplogroups, and grey corresponds to second layer lineages/ haplogroups.

We find that calibrating the tree in this way reveals a correspondence between the root height of putative second layer *Mtb* lineage 2 (48.9Ky; HPD: 41.4–57.1Ky) and Y-chromosome haplogroup NO (49.8Ky; HPD: 47.8–51.8Ky). We also observed *Mtb* Lineage 1 to display similar qualitative characteristics to putative first layer Y-chromosome haplogroups C, D and K2b1. These lineages displayed early ‘lineage proliferation’ phases, in which the number of lineages initially expands, followed by a period of little subsequent increase (Fig. 5a).

By comparing the tree structures quantitatively using the ‘lineages through time’ metric, we also observe correspondence between the Y-chromosome and *Mtb* lineages thought to derive from first and second layer groups. Putative first layer lineage counts (*Mtb* L1, Fig. 5b; Y-chromosome lineages C, D and K2b1, Fig. 5c) gradually increase, while putative second layer lineage counts (*Mtb* L2, Fig. 5b; Y-chromosome lineage NO, Fig. 5c) expand dramatically closer to the tips of the trees. We also performed a more formal comparison by deriving rates of change from these trajectories^38^, and contrasting values obtained for putative first and second layer lineages. This approach revealed putative second layer lineages to be characterised by growth rates which were consistently faster than their first layer counterparts (Wilcoxon tests for trajectories L1 vs L2, NO vs C, NO vs D and NO vs K2b1; all *p* values < 0.038; Supplementary Note 4; Supplementary Fig. 23). This trend is particularly pronounced when considering the timeframe spanning the Neolithic period (10Kya) until the present (all *p* values < 2.62 × 10^−6^).

In line with the divergence ages we estimate, our inferred substitution rate (2.16 × 10^−9^s/s/y) is intermediate between the various estimates of Comas et al. (2013)^38^ (9.42 × 10^−10^ to 1.66 × 10^−8^), but slower than estimates derived from tip dating using contemporary samples^80^ (around 1×10^−7^). As detailed subsequently (see Discussion), this magnitude of substitution rate differences is typical of other pathogens when applying these two techniques over comparable timescales^81,82^. We also discuss the plausibility of the inferred substitution rate given available estimates from *Mtb* during latency^83,84^, and when considering patterns of rate heterogeneity within similar bacteria^85,86^. Although recent studies have implemented methods for modelling variation in substitution rates over different timescales^70,76^, the lack of additional high-confidence internal calibration points within the *Mtb* tree precluded us from applying these approaches with certainty.

In sum, these data draw a link between *Mtb* lineage 1 and Y-chromosome haplogroups C, D and K2b1 and the initial first layer hunter-gatherer presence in East Asia, and between *Mtb* lineage 2 and the second layer.

### Novel deeply rooted *Mtb* L1 sublineages in Eastern Indonesia and PNG

We now describe a final line of suggestive evidence to link patterns of *Mtb* variation with the Two Layer model, by documenting a relevant aspect of lineage 1 phylogeographic diversity. Knowing that the Indigenous populations of Oceania and Eastern Indonesia represent largely unadmixed descendants of the first layer of peopling, we aimed to characterise the diversity of *Mtb* lineage 1 in this region. We reason that the identification of deeply rooted lineage 1 sublineages within these populations would support the association of lineage 1 and the first layer dispersal into the region.

**Fig. 6:**
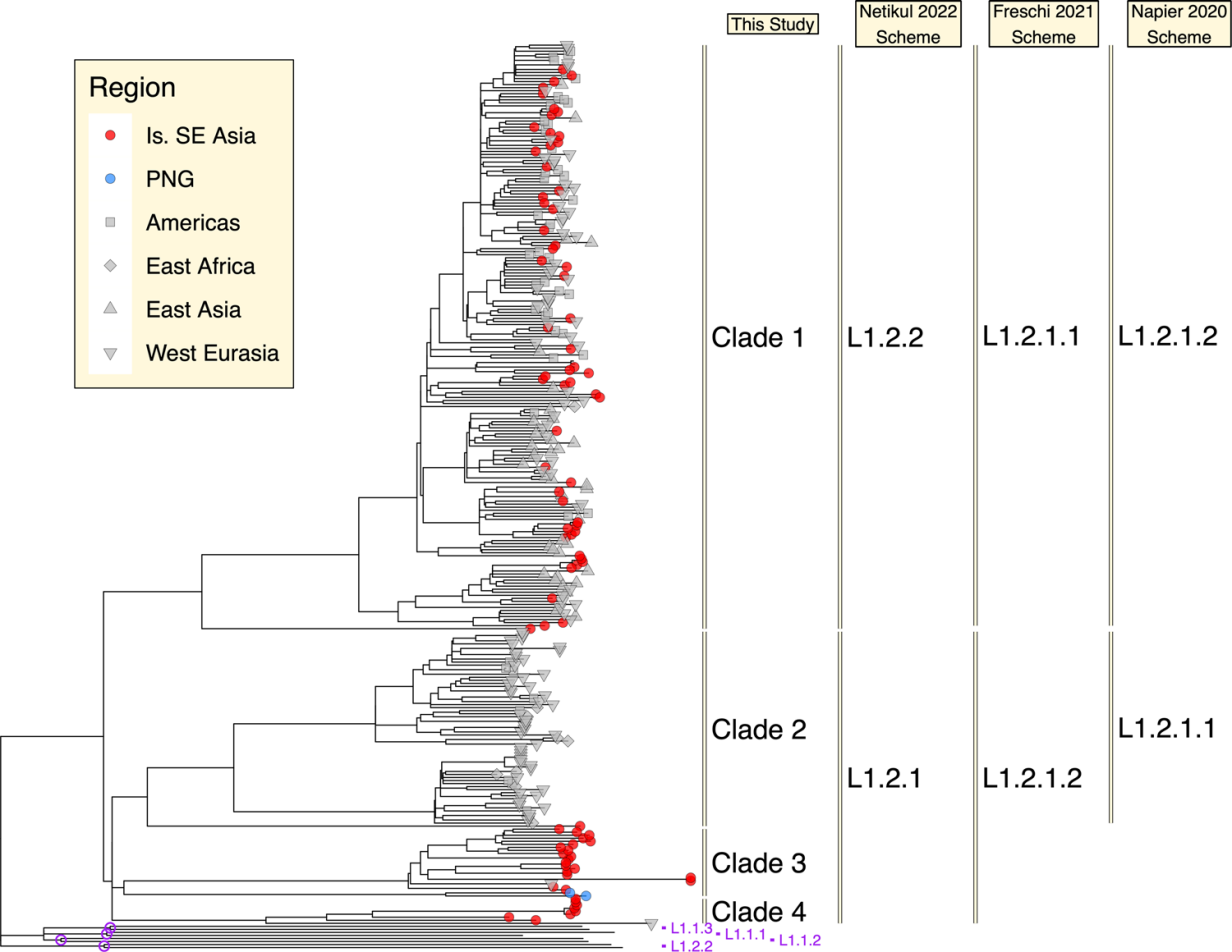
Phylogeny of *Mtb* lineage 1.2.1 isolates from three prior studies^79, 87, 88^. Tip point colours and shapes correspond to geographical sampling locations, with red and blue circles designating Island Southeast Asia and PNG respectively, and grey shapes representing all other regions. L1.2.1 clades were labelled according to three alternate nomenclature schemes for additional context. Isolates representing the single deepest split in each of the remaining L1 sublineages are included. Each of these sublineage diversification events is marked with a purple circle, with purple tip labels indicating the sublineage of these isolates.

We assembled a dataset of isolates from lineage 1.2.1, which is the predominant L1 sublineage in Island Southeast Asia, from three previous studies^79,87,88^. We chose to downsample the high number of isolates belonging to the well characterised ‘Manila’ and ‘Nonthaburi’ clades. To assess the relative divergence point of L1.2.1 with respect to other sublineages of L1, we incorporated isolates (where available) representing the single deepest split within the remaining L1 sublineages: L1.1.1, L1.1.2, L1.1.3 and L1.2.2^87^. We inferred a phylogeny, and labelled L1.2.1 sublineages according to the nomenclature of three available schemes, but here refer to them as Clades 1 to 4 (Fig. 6).

Figure 6 shows the L1.2.1 sublineage to be characterised by a series of deep splits, each of which results in descendant isolates sampled from either Island Southeast Asia or PNG. Clade 1, which includes the common Manila and Nonthaburi genotypes, occurs most commonly in Southeast Asia and shows evidence of recent expansion to other global regions. The most deeply rooted isolate from this clade was sampled from Borneo.

Clades 2, 3 and 4 form a monophyletic group, which diversified very soon after the origin of the L1.2.1 sublineage. Clade 3 was isolated only from patients in East Timor and PNG, and a single European patient^79,87^. The split between East Timorese and Papuan isolates within this clade was extremely deep. Clade 2 was found in patients from both Europe and Africa, but also possessed a deeply rooted isolate sampled from Borneo. Clade 4, a sublineage documented for the first time here, was isolated from 8 patients from Indonesia and one from Europe. The under-representation of Island Southeast Asia and Oceania in studies of *Mtb* genomics may mean that other deeply rooted L1.2.1 lineages have yet to be sampled.

We considered the relative divergence point of L1.2.1 sublineages and found them to match those of other sublineages of L1, implying that *Mtb* has been present in Island Southeast Asia for as long as it has been present in other regions around the rim of the Indian Ocean. We therefore propose that the strong regional structure and deep divergence of a sublineage of L1 in Oceania and Eastern Indonesia may couple this *Mtb* lineage with the human populations which arrived here as part of the first layer of peopling.

**Fig. 7:**
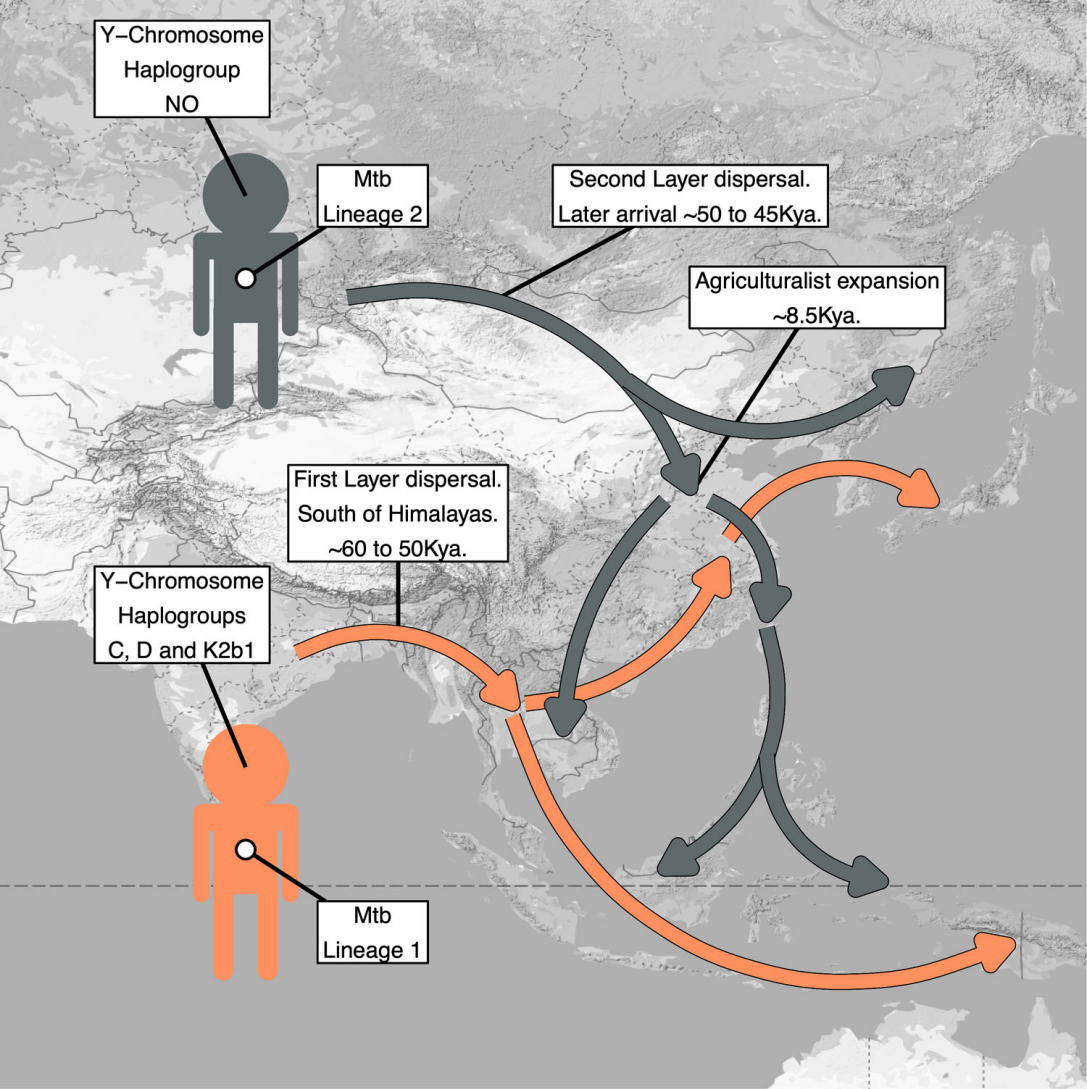
Our proposed model of human-*Mtb* co-expansion, including population dispersal trajectories, and the predominant *Mtb* and Y-chromosome lineages they carried. See Matsumura et al. (2019)^2^ for a corresponding diagram based off anthropometric data. Map was sourced from Google Maps using the using the ‘get_googlemap’ function of ggmap^129^.

Synthesising the above data, we present in Fig. 7 our model of human-*Mtb* coexpansion as it pertains to the Two Layer hypothesis.

## Discussion

We have conducted a joint analysis of the spatial and temporal dynamics of human Y-chromosome and *Mtb* sequences in East Asia, and synthesised a coevolutionary model compatible with the Two Layer hypothesis. Under this model, we propose Y-chromosome lineages C, D and K2b1 to have arrived in East Asia via an initial southern coastal route migration, by human populations which also carried *Mtb* lineage 1 (Fig. 7). We suggest the Y-chromosome NO clade to have arrived via a northern route expansion across Eurasia, carried by human populations carrying *Mtb* lineage 2 (Fig. 7).

Haplogroups C, D and K2b1 share the phylogenetic characteristics of deep roots and sparse internal branches, and are enriched in the traditionally hunter-gatherer human populations of East Asia. These haplogroups do not show evidence of extreme population growth associated with the development of agriculture and show sparse and scattered spatial frequency distributions. The diversity of the most prevalent of these haplogroups, haplogroup C, decreases dramatically when moving westwards into inland Eurasia.

We have inferred *Mtb* lineage 1 to also be characterised by a deep divergence time and sparse internal branches, and to display spatial frequency and diversity characteristics most compatible with a southern entry route. We propose that the presence of deeply rooted and regionally diverse lineage 1 sublineages in PNG and Eastern Indonesia also supports the association of this *Mtb* lineage with this layer of peopling. Prior archaeological studies have documented representatives of these populations to have settled soon after the initial dispersal of humans out of Africa^89^. This conclusion is reflected in the phylogenetic characteristics of both Y-chromosome and mitochondrial lineages amongst Indigenous Oceanians, which are deeply diverged from other worldwide lineages, and which undergo an early and rapid diversification from one another approximately ~45Kya^90^. The mirroring of these characteristics in the *Mtb* lineage 1 phylogenetic tree supports its association with the initial hunter-gatherer populations of the region.

Conversely, the temporal dynamics of the NO clade suggest it to have expanded at a timepoint concurrent with the development of agriculture, and to exhibit highest frequencies in Chinese populations. The connection of this lineage with this cultural development is further supported by its underrepresentation in traditionally hunter-gatherer populations in East Asia, and its strong enrichment in nearby agriculturalist groups. A dispersal trajectory of the NO lineage north of the Himalayas can be inferred from the extremely low diversities of this haplogroup in South Asian populations. We also note that the presence of a basal K2ba* haplogroup, which is a precursor to the NO lineage, retrieved from Ust'-Ishim in central Eurasia ~40Kya also supports a northern route origin of this lineage^91^.

We propose the phylogenetic characteristics of the NO clade to mirror those of *Mtb* lineage 2, as both NO and L2 display similar origin points relative to first layer lineages, and both undergo rapid phases of lineage expansion at similar phylogenetic depths. We infer the region of highest frequency and diversity of *Mtb* lineage 2 to be central East Asia, and find low frequencies and diversities of this lineage in regions along the proposed southern entry route.

There are some limitations in the scope of the presented model which, due largely to data availability, deals only with the populations of East Asia, and not with groups from more westerly regions. It is evident from both our analysis and prior surveys^37,92^, that *Mtb* lineage 1 has a strong presence in South Asian populations, particularly southern India, and that certain L1 sublineages are geographically restricted to this region^79,87^. Conducting a more detailed analysis of human and *Mtb* coevolution in South Asia, although not undertaken here, will therefore be necessary to fully account for the patterns of spatial frequency and diversity of lineage 1 depicted in Fig. 4a.

While we did not undertake this analysis, we do observe congruent features in the *Mtb* and Y-chromosome composition of South Asian populations. Prior studies have shown Indian populations to be modelled as mixtures of Ancestral Northern Indian (ANI) and Ancestral Southern Indian (ASI) ancestry, the latter component of which is linked to Indigenous first peoples of East Asia such as the Andamanese^93^. Populations enriched for ASI ancestry, which are predominantly located in southern India, possess high frequencies of haplogroup H^44^, which we note to possess the phylogenetic characteristics of deep divergence times and limited population growth^46^. Considering south Indian populations are also enriched for *Mtb* lineage 1^94^, we tentatively propose Y-chromosome haplogroup H to be associated with the initial hunter-gatherer presence in South Asia, and to be associated with this *Mtb* lineage.

We have also emitted some Y-chromosome and *Mtb* lineages from our model due to their low frequencies or lack of available data. One notable omission is *Mtb* lineage 4, which is present in low to moderate frequencies in some East Asian populations^35,95,96^. We omitted this lineage, as our focus was on *Mtb* and Y-chromosome lineages originating in East Asia, hence why Y-chromosome haplogroups Q, R, F* and H were not analysed extensively. As prior phylogeographic studies have shown the majority of L4 sublineages to display European origin points^40^, and a history of multiple introductions into Southeast Asia^35^, we chose to exclude this lineage from consideration. Interestingly, however, a small number of L4 sublineages are geographically restricted to East Asia, with highest frequencies in Chinese populations (L4.4 and 4.5^95,97^). More detailed analysis may reveal these to be minor lineages associated with the second layer of peopling, or later demographic processes outside the scope of this investigation. For context, lineage 4 was only observed at a frequency >20% in the single Chinese cohort included in this analysis^95^. Spatial frequency interpolation also revealed low L4 frequencies in East Asia (Supplementary Note 3; Supplementary Fig. 20).

We wish to note that our model of historic human and *Mtb* co-expansion is one which infers a Paleolithic origin of contemporary *Mtb* variation. Recent molecular dating studies have inferred substitution rates which favour a Neolithic origin of *Mtb*, likely within the past 6000 years^98–100^, as opposed to the past 60,000^38,39^. We highlight three factors to note when considering Paleolithic vs Neolithic *Mtb* emergence theories.

Firstly, none of the recent studies inferring substitution rates on *Mtb* used genomes older than 350 years^98,99^, and the three most ancient *Mycobacterium tuberculosis* complex (MTBC) isolates analysed, incidentally all belonging to *M.* *pinnipedii*, were <1Ky old^100^. The phenomenon of ‘time dependency’ in substitution rate estimates is well documented in viruses and bacteria, with studies documenting systematic over-estimation of mutation rates when calibrating based on more recent samples^67,73,101^.

We note the differences in substitution rates inferred from our analysis (2.16 × 10^−9^) relative to tip dating^80^ (~1 × 10^−7^), are roughly within the ranges established for other pathogens^81,82^. Hepatitis B virus substitution rate estimates vary around two orders of magnitude when comparing human co-divergence calibration with tip dating^81^, and foamy virus substitution rates vary up to four when considering primate species co-divergence^82^. Available data for bacteria, although more limited, has suggested an order of magnitude difference in substitution rates inferred from contemporary samples vs samples approximately 1Ky old^67^. A recent review collating estimated mutation and substitution rates in *Mtb* specifically has also concluded the presence of a time-dependence effect^102^, although high quality DNA from more ancient *Mtb* genomes will be required to properly gauge its magnitude^80^.

Secondly, prior studies have proposed that the mutation rate of *Mtb* may not be static across time^65^. The mutation rate quantifies the instantaneous speed of mutation accumulation within *Mtb* bacteria, and therefore differs from the substitution rate, which describes a rate inferred using a temporal phylogeny^103^. Given the drastic transition in lifestyles from hunter-gatherers to agriculturalists in the Neolithic, it has been postulated that the *Mtb* pathogen also underwent a shift in life history strategy^65,66^. It is possible that in Paleolithic times, *Mtb* genomes spent more time in a latent state, and accumulated mutations at a slower rate than in the present day^65^.

Available data from other bacteria supports dormancy-related levels of rate variation which may be capable of explaining the substitution rate we infer for *Mtb*. Cui et al. (2012)^85^ find that branches of the *Y. pestis* phylogeny, a bacterial species with a similar genome size and general mutation rate to *Mtb*, can vary in substitution rates by 40-fold, from ~1.3 × 10^−7^ to 3.1 × 10^−9^. They attribute this variation to the bacteria’s ability to enter a dormant phase between epidemic periods. Similar substitution rates and levels of rate heterogeneity are also reported by Spyrou et al. (2019)^86^ for *Y. pestis*. It is plausible that the combination of latency and the relatively stronger effect of purifying selection in *Mtb* (dN/dS = 0.6^104^); relative to *Y. pestis* (dN/dS = 0.9^85^); could explain the slow long-term substitution rate we infer.

In addition, data describing the mutation rate in human *Mtb* infection during latency supports a slowdown effect^84^, and mutation rates broadly consistent with Paleolithic emergence theories during long-term latency. When analysing samples with an apparent latency period of around 20 years, Colangeli et al. (2014)^83^, for instance, propose a mutation rate of 7.01 × 10^−9^. It should be noted, however, that these studies are sparse, have low sample sizes, and have produced conflicting results^105^. Collectively though, these data provide reasonable justification to credit the likelihood of a broad range of possible substitution rates for *Mtb*.

A final form of data which should be taken into account when considering *Mtb* emergence scenarios comes from the fields of paleomicrobiology and ancient DNA^106–108^. These studies, which amplify *Mtb* sequence motifs, have produced evidence consistent with the presence of the pathogen in samples from Neolithic Israel^109,110^, Syria^111,112^, Germany^113^, Hungary^114–116^, Egypt^117^ and Britain^118^, dating up to an estimated 10.8Kya. Multiple studies have also detected the TbD1 deletion^110,113,117,118^, implying that evolutionarily modern lineages were present at these timepoints. Further scrutiny will be required to verify these findings, and claims addressing the impact of mycobacterial contaminants, which have been previously brought into question^119,120^.

To facilitate a comparison of possible *Mtb* emergence theories, we present in Supplementary Table 1 the substitution rates and node ages inferred using our model and several alternate models based on either tip or node calibration. A key line of evidence in support of the Paleolithic emergence scenario we propose is that *Mtb* node ages appear to correspond to key demographic events in human history.

For instance, when calibrating the *Mtb* phylogeny so that the age of putative first layer lineage (L1) matches that of first layer Y-chromosome lineages, we also see a strong similarity between the inferred ages of putative second layer lineages and haplogroups (NO and L2; both ~49Kya). This date is also consistent with the date proposed by Matsumura et al. (2019)^2^ for the arrival of this second layer dispersal on the basis of archaeological data (45Kya).

Our model also predicts a coalescence point for the entire MTBC that is consistent with the deepest splits of human Y-chromosome lineages within the African continent. *Mtb* lineages 1 and 2 coalesce 112.4Kya (HPD: 94.1–131.9Kya), and prior phylogenetic analyses show the split of *M.* *africanum* lineages (L5 and L6) to be roughly 10% older than this (Comas et al. 2013)^38^. Aside from the rare West African A00 haplogroup discovered after extensive consumer genetic testing (divergence time: 250Kya), the divergence times of all major Y-chromosome haplogroups fall between roughly 60 and 190Kya^27,121^. The localisation of the most deeply rooted *Mtb* lineages to the African continent, with phylogenetic depths comparable with human uniparental lineages supports an out-of-Africa co-dispersal scenario for the pathogen^22^.

Paleolithic emergence scenarios also receive support from the geospatial dynamics of *Mtb* lineages, as well as their clinical characteristics and life history strategies. The ‘evolutionarily modern’ lineages 2, 3 and 4 show expansion centres corresponding to major centres of agriculture in Asia and Europe, and levels of transmissibility suggesting adaptation to high population sizes^39,122,123^. By contrast, the less virulent and transmissible lineage 1 has been hypothesised to be adapted to hunter-gatherer human populations^66^. Our model therefore provides an explicit demographic explanation for the distribution and dynamics of lineage 1, by linking it to the original hunter-gatherer populations of East Asia.

Available tip calibration scenarios, on the other hand, don’t provide node ages as immediately parsimonious with known human demographic events, or at least, no single model linking these dates and events has been proposed to date. This, however, does not necessarily mean that *Mtb* node ages and evolutionary dynamics dated using tip calibration are incompatible with Paleolithic emergence theories. As others have hypothesised^67,80^, substitution rates inferred using tip calibration may be appropriate for dating relatively shallow nodes within the tree, but not for the deep nodes we focus on in this study. The combination of ancient DNA, and research into the demographic factors underpinning the spread of *Mtb* lineages will clarify this matter.

The final limitation of this analysis we note is that inferences of admixture proportions in human populations can be confounded when analysing patterns of uniparental genetic variation. Biases in the relative contribution of male vs female lineages from a given ancestry group present themselves when considering most large-scale admixture events in modern times. These include, for instance, the events associated with the colonisation of the Americas^124^ and Greenland^125^, as well as the initial peopling of Polynesia^126^ and Madagascar^127^. We caution that Y-chromosome lineages from the first layer of peopling may have contributed in disproportionately low frequencies to the present-day genetic constitution of East Asian populations. This factor may explain the finding that putative first layer *Mtb* lineages are comparatively more numerous than first layer Y-chromosome lineages across many contemporary East Asian populations and show signs of more pronounced lineage expansion (Fig. 5). It follows that exploration of the dynamic of the Two Layer model from the perspective of maternal lineages may yield different inferred ancestry proportions and should be an avenue of future research.

## Methods

### Ethics declaration

All datasets included in this analysis were retrieved from prior peer reviewed publications. No datasets containing identifiable information were included. All studies selected document receiving informed consent and/ or ethical approval from relevant organisation(s).

### Y-chromosome haplogroup frequency analysis

Y-chromosome haplogroup frequencies were collated from previous studies, described in greater detail in Supplementary Note 1. We ensured that individuals were genotyped to a high enough degree of resolution to preclude the possibility of spuriously classifying NO*, L, or T lineages as K-M526*^42^. The studies from which the frequencies of Y-chromosome haplogroups in traditionally hunter-gatherer populations were summarised are discussed in Supplementary Note 1, along with the rationale for the comparison of each non-Indigenous group.

Spatial interpolation of haplogroup frequencies was carried out using the kriging procedure. This process includes fitting a variogram describing the relationship between geographic distance and haplogroup frequency differences for the chosen haplogroup, before interpolating the frequency of this haplogroup across a spatial surface. These steps were completed using various functions of the gstat package in R^128^ and visualised using ggmap^129^. We provide an illustration of the concordance between observed and interpolated haplogroup frequencies in Supplementary Note 1 (Supplementary Figs. 4–7), and also present kriging plots (Supplementary Note 1, Supplementary Figs. 8–11) for additional Y-chromosome haplogroups. Also included and separate kriging plots for haplogroups N and O (Supplementary Figs. 12 and 13).

### Y-chromosome haplogroup diversity analysis

Y-chromosome STR profiles were obtained from previous studies^29,31,59,60,130–137^. Six short tandem repeats which were genotyped across all datasets were used for diversity calculations (DYS389I, DYS389II, DYS390, DYS391, DYS392 and DYS393). Minor discrepancies in the way the DYS389II repeat length was recorded between studies were corrected for, and haplotype diversity was measured using the approach of Nei (1987)^138^ using custom Unix scripts.

### Y-chromosome phylogenetics

Genotypes called across the Y-chromosome for samples from the HGDP^46^ were restricted to biallelic SNPs and used to produce a fasta alignment of all variable sites. The regions of the Y-chromosome analysed were further restricted to the 10.4 Mb shown to be amenable to phylogenetic research^71^. BEAST^139^ was used to construct a Y-chromosome phylogeny describing the relationship between all lineages in this dataset falling into clades C, D, K2b1 and NO, which were identified from data presented in the supplementary material of Bergstrom et al. (2020)^46^. The BEAST parameters included a GTR model of nucleotide substitution, an uncorrelated lognormal relaxed clock to model rate heterogeneity between branches, a Coalescent Bayesian skyline tree prior, and a Y-chromosome specific mutation rate derived from the analysis of Fu et al. (2014)^140^. This rate was corrected to account for the fact that the alignment was restricted to variable sites only. BEAST was run in five independent replicates of 10,000,000 iterations, sampling every thousandth, before combining with LogCombiner. A Maximum Clade Credibility tree was produced using TreeAnnotator. Key splits in the phylogeny received high posterior support and were in fine agreement with those of Bergstrom et al. (2020)^46^ on the basis of both branching pattern and node age. Bayesian skyline estimates of effective population size were obtained after running BEAST on samples from each haplogroup individually for 30,000,000 iterations.

### *Mtb* frequency and diversity analysis

Kriging of *Mtb* lineage frequencies was carried out using an identical approach to that described above for Y-chromosome haplogroups. We used the dataset of Wiens et al. (2018), which describes global lineage frequencies, to perform kriging and calculated spoligotype diversity using data from SITVIT2^63^. We calculated diversity values for spoligotype families representative of lineages 1 (all ‘EAI’ clades) and 2 (‘Beijing’ clades) at each sampling location using the same diversity index as described for Y-chromosome haplogroups. We excluded isolates which were sampled from immigrants, and populations represented by a low number of isolates (*n* = 40) for each lineage. As per the rationale of prior studies^37,79^, we have not shown diversity values for countries in Western Europe or Australia, however frequency values for these countries are still shown. Plots showing the concordance of observed and interpolated *Mtb* lineage frequencies for lineages 1, 2, 3 and 4 are shown in Supplementary Figs. 17–20.

### *Mtb* phylogenetics—lineage 1 and 2 analysis

We collated *Mtb* WGS data from prior studies which conducted random sampling within East Asian populations, and which weren’t enriched for drug resistant isolates^35,95,96,141–143^. To obtain an unbiased comparison of the dynamics of *Mtb* and Y-chromosome evolution, we down-sampled isolates from each of these studies so that the total number of *Mtb* lineage 1 and 2 isolates matched the number of Y-chromosome sequences from which the phylogeny was inferred. Fastq files for all isolates were downloaded from the Sequence Read Archive, and were quality trimmed using cutadapt^144^. Reads were then aligned to the H37Rv reference genome using BWA^145^, filtered for duplicates using Picard^146^, and subjected to variant calling using Pilon^147^. Poor quality and ambiguous variant calls were then set to missing, and a lineage was assigned to each sample using ‘fast-lineage-caller’^148^ and the nomenclature scheme of Coll et al. (2014)^149^. All per-sample vcf files were then merged, and both per-site and per-sample missingness filters were applied using bcftools^150^. Finally, variant calls were restricted to those in uniquely mappable regions of the *Mtb* genome before phylogenetic inference, using the coordinates of Brynildsrud et al. (2018)^40^.

As described in the Main Text, we used the phylogenetic technique of ‘node calibration’^68–71^ to scale our *Mtb* tree using signatures of human-*Mtb* co-divergence. We initially inferred an *Mtb* phylogeny from a sequence alignment incorporating all L1 and L2 isolates, assigning a prior distribution over the age of the L1 root. For this prior, we used a lognormal distribution with mean value of 56.5Ky (the average age of Y-chromosome haplogroups C, D and K2b1) ± 5Ky. Our phylogenetic parameters were otherwise the same as used for the Y-chromosome analysis, including the use of an uncorrelated lognormal relaxed molecular clock. We again ran BEAST in five independent replicates, before combining log files using LogCombiner. We obtained a substitution rate from this log file (2.16 × 10^−9^s/s/y; HPD: 1.92 × 10^−9^ to 2.40 × 10^−9^) after correcting for the fact that only polymorphic sites were included in the alignment. The resulting Maximum Clade Credibility tree is presented in Fig. 5a. To generate the accompanying ‘lineages through time’ trajectories for each lineage (Fig. 5b), we ran BEAST independently for 50,000,000 iterations on alignments which had been subset to include only L1 and L2 sequences respectively. We calibrated these trees by assigning the same prior distribution to the L1 root as described above (56.5Ky ± 5Ky), and setting a prior on the L2 root to match the age inferred in the tree in Fig. 5a (48.9Ky ± 5Ky).

### *Mtb* phylogenetics—lineage 1.2.1 analysis

To infer a phylogeny representative of known lineage 1.2.1 diversity, isolates used in the investigations of Menardo et al. (2021)^79^ and Netikul et al. (2022)^87^ were collated. As detailed sublineage data was available for the isolates from Netikul et al. (2022)^87^, we were able to preferentially down-sample isolates from Clade 1 (Fig. 7; designated L1.2.2 by Netikul et al.) to 5 representative lineages. All isolates documented by Menardo et al. (2021)^79^ were include in our analysis, and we also included all L1.2.1 isolates from the study of Meumann et al. (2021)^88^. The protocol for downloading and calling variants on these isolates was identical to that used above for the *Mtb* ‘lineage 1 and 2’ dataset, with the only difference being an additional adapter inference and filtering step^151^, and appropriate pipeline adjustments for samples with single end reads. Representative isolates from lineages 1.1.1.1, 1.1.1, 1.1.2, 1.1.3, 1.2.2 and 5 were also downloaded and processed in the same manner as other samples. We used data presented by Netikul et al. (2022)^87^ to identify isolates representing the deepest split in each L1 sublineage. We inferred a phylogeny using RAxML^152^ using a GTR substitution model, an L5 outgroup, and annotated lineages according to various nomenclature schemes^87,148,153^.

### Statistics and reproducibility

All studies from which data was drawn are referenced in the text. Only studies which used random sampling regimes (as opposed to preferentially selecting samples belonging to a particular Y-chromosome haplogroup, or with a particular drug resistant status) were used. Samples were only filtered on the basis of sequencing data quality. For the *Mtb* phylogenetic analyses [‘lineage 1 and 2 analysis’ (Fig. 5) and ‘lineage 1.2.1 analysis’ (Fig. 6)] that involved random sub-sampling, lists of the isolates that were retained are provided in Supplementary Data 1.

Multiple independent replicates of each BEAST phylogenetic analysis were run to check for consistency, and skyline trajectories were replicated after running BEAST with different parameter values (see Supplementary Note 2). A *p* value threshold of 0.05 was used for the statistical tests reported in Supplementary Notes 3 and 4.

### Reporting summary

Further information on research design is available in the Nature Portfolio Reporting Summary linked to this article.

### Supplementary information

Peer Review File

Supplementary Information

Description of Additional Supplementary Files

Supplementary Data 1

Reporting Summary

## Data Availability

All data analysed in this paper was drawn from previously published studies, as specified in the text and in Supplementary Data 1. Raw lineage through time values depicted in Figs. 2c, 5b and 5c are provided in Supplementary Data 1. The proportions depicted in the pie charts in Fig. 1 are listed in Supplementary Note 1, section iv.
