# Supplementary material for: Parallel signatures of Mycobacterium tuberculosis and human Y-chromosome phylogeography support the Two Layer model of East Asian population history
Source: Commun Biol. 2023 Oct 13;6:1037. doi: 10.1038/s42003-023-05388-8 (PMC10575886; doi:10.1038/s42003-023-05388-8)
Supplement: Supplementary file 2 — Supplementary Information [file 42003_2023_5388_MOESM2_ESM.pdf]

Supplementary Note 1. Y-Chromosome Haplogroup Frequency Analysis

i) Dataset Compilation

Y-chromosome haplogroup frequency data was collated from previously published studies[1,2,3,4,5]. We ensured Y-chromosomes were genotyped to the current gold-standard in phylogenetic resolution[6], which includes markers defining haplogroups L, T and NO*[6]. Only populations for which precise latitude and longitude coordinates could be retrieved from the study, or closely approximated using maps presented in the study, were used (Supplementary Figure 1).

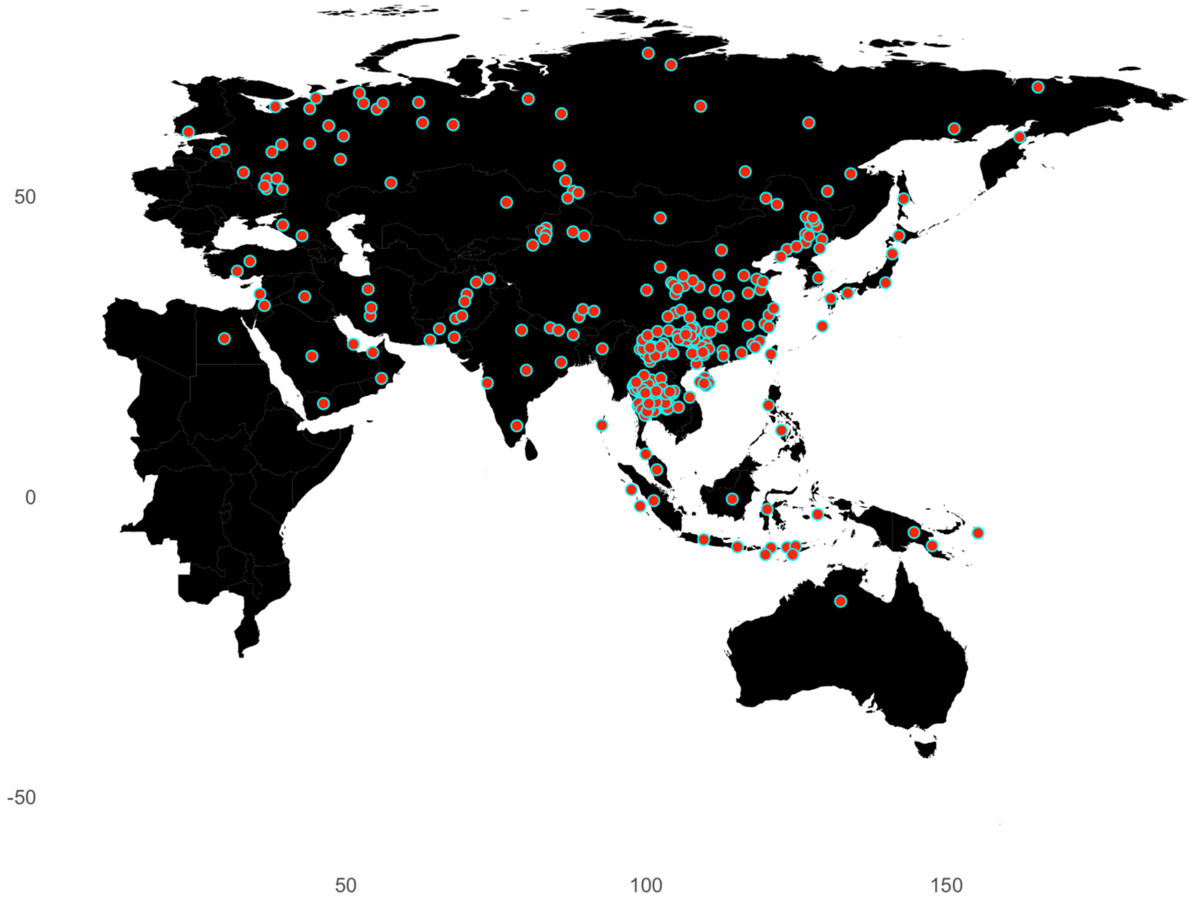

Supplementary Figure 1) Map showing locations of populations used for the spatial frequency analysis.

ii) K2b1 spatial frequency interpolation plot presented in Figure 3d

One of the haplogroups of interest to this study is K2b1. This haplogroup is defined by the markers P397 and P399 and constitutes a substantial proportion of Y-chromosome haplogroups in the populations of Oceania[7]. These two markers were discovered relatively recently and therefore weren't genotyped in all of the studies analysed here.

For the spatial frequency interpolation of lineage K2b1 (Figure 3d), only haplotypes which could be unambiguously assigned to haplogroups K2b1 were used, designated by the presence of downstream markers for haplogroups M and S[5]. This is a conservative choice and has minimal effect on the interpretation of this figure.

Karafet et al. (2015)[7] demonstrate through a survey of over 7,000 K-M526 Y-chromosomes that the vast majority of Island Southeast Asian K-M526* haplogroups, which don't belong to haplogroups M or S, fall into the K2b1 clade. The spatial frequency estimates of the K2b1 haplogroup depicted in Figure 3d are therefore likely to be slightly underestimated in Island Southeast Asia.

Importantly, we find K2b1 to be highly unlikely to occur outside Island Southeast Asia. In our survey, there were 8 other populations from the sample of 298 populations which possessed K* haplogroups, most in the Middle East, and most with frequencies of below 1%. These lineages are likely to represent other K* haplotypes not belonging to K2b1, as Karafet et al. (2015)[7] show K2b1 to be completely absent from the region.

iii) Main Y-chromosome lineages in East Asian populations

We restricted the focus of our Y-chromosome analysis to the four predominant East Asian Y-chromosome lineages. Our rationale for selecting these lineages is based on haplogroup frequencies from our survey, and data from prior studies which have assigned geographical provinces and origin points to haplogroups. To assess haplogroup frequencies we drew a geographical transect at longitude 90 and considered all populations to the east of this boundary (Supplementary Figure 2). This transect runs through the apex of the Bay of Bengal and approximates the division between South and East Asia. We calculated haplogroup frequencies as the mean value across all populations retained.

Across all East Asian populations, we found haplogroup C to attain an average frequency of 11.6%, haplogroup D 4.8%, haplogroups K2b1/K* 4.5%, and haplogroup NO 72.7% (Supplementary Figure 3). All other Y-chromosome haplogroups had frequencies of less than 1%, aside from haplogroup R with a frequency of 2.5% (Supplementary Figure 3). As demonstrated through previous studies, R has a West Eurasian origin, and is present only on the fringes of East Asia, permeating the border of South Asia and Siberia[8,9] (Supplementary Figure 8).

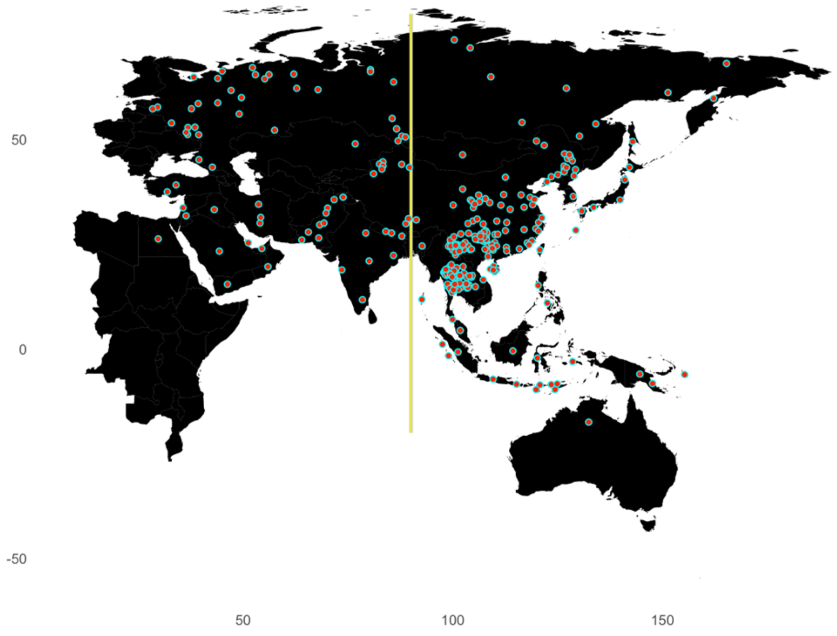

Supplementary Figure 2) Map showing geographic transect used to define East Asian populations for the analysis of Y-chromosome haplogroup frequencies. All populations to the east of the yellow line, drawn at longitude 90, were considered 'East Asian' for the purposes of this analysis.

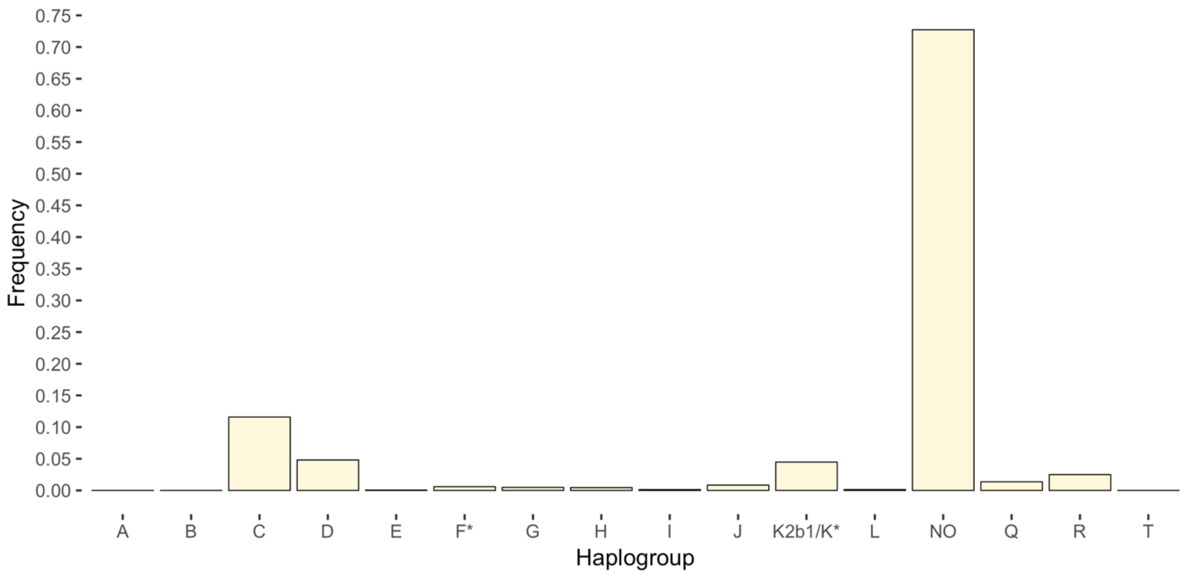

Supplementary Figure 3) Average haplogroup frequencies across all 'East Asian' populations.

iv) Haplogroup Frequency Enrichment in First Layer Populations

We summarised data presented in previous genetic studies to illustrate the enrichment of Y-chromosome haplogroups C, D and K2b1 in the traditionally hunter-gatherer first layer populations of East Asia. We contrasted haplogroup frequencies in each of these populations with those of a nearby non-Indigenous group. As was the case for the Burmese[10], the Japanese[11], and the Philippines[12], multiple nearby populations were pooled to maximise sample size, as detailed below.

Some of the studies included in this survey were not genotyped to a high enough degree of resolution to unambiguously assign K* lineages to the K2b1 clade. To reflect this uncertainty these haplogroups were designated as K2b1/K*. The analysis of Karafet et al. (2015)[7] suggests that the vast majority of Oceania and Island Southeast Asian K* lineages do, however, fall into clade K2b1. Specific details of each chosen study, the precise genotyping scheme, and any additional notes are detailed below.

Onge and Burmese – Y-chromosome haplogroup frequency estimates for the Onge were summarised from the data of Thangaraj et al. (2003)[13], who find all 23 male samples to belong to haplogroup D (Frequencies: C - 0, D - 1, K2b1 - 0, NO - 0, Other - 0). For the Burmese reference population, a total of 114 Y-chromosome haplogroups from Peng et al. (2014)[10] were collated (Frequencies: C - 0.035, D - 0.035, K2b1 - 0, NO - 0.76, Other - 0.18).

Papuan and Austronesian – Papuan Y-chromosome frequencies from a total of four populations described by Kayser et al. (2006)[14] were included (n=247). The majority of Papuan samples belonging to haplogroup K (n=183/200) could be unambiguously assigned to the K2b1 clade, due to the presence of markers belonging to either the M or S clades. It is

likely that the remaining 17 K individuals also fall into lineage K2b1, as Karafet et al. (2015)[7] documented all 40 K(xNO) Papuan samples to belong to K2b1 sub-lineages in their analysis. To express this degree of uncertainty, however, this lineage was labelled K2b1/K* in Figure 1 (Main Text) (Frequencies: C - 0.16, D - 0, K2b1 - 0.81, NO - 0.024, Other - 0.004).

Given the other main source of ancestry in present day Oceanic populations is derived from Austronesian migrants[15], we chose a representative Taiwanese group to contrast Papuan haplogroup frequencies with. Taiwan is widely documented as the origin of the Austronesian expansion[16,17,18]. To represent these groups, we used a sample of 48 Taiwanese Y-chromosomes from Karafet et al. (2010)[5] (Frequencies: C - 0.021, D - 0, K2b1 - 0, NO - 0.98, Other - 0). Importantly, the conclusions of this analysis remain unchanged when alternate Oceanic reference populations are used, such as 'Micronesia', from Zhong et al. (2011)[1].

Ainu and Japanese – To obtain haplogroup frequency estimates in Ainu populations, data from Tajima et al. (2004)[19] and Hammer et al. (2005)[20] was summarised. Together, this represents a summed sample size of 20 individuals (Frequencies: C - 0.15, D - 0.85, K2b1 - 0, NO - 0, Other - 0). To obtain estimates of haplogroup frequencies in a cosmopolitan Japanese population, the Nonaka et al. (2007)[11] dataset was used. This dataset contains Y-chromosome haplogroups for 263 Japanese individuals from various provinces (Frequencies: C - 0.053, D - 0.39, K2b1 - 0, NO - 0.55, Other - 0).

Philippine hunter-gatherer and Philippine – Data from a pooled sample of 180 Indigenous Philippine samples described by Delfin et al. (2011)[21] was summarised. The enrichment signal of putative 'first-layer' Y-chromosome lineages is more pronounced if only groups with minimal degrees of non-Indigenous ancestry such as the Aeta and Agta are considered[21]. The study of Delfin et al. (2011)[21] does not genotype markers P397 and P399 which define lineage K2b1. To express this uncertainty, all K* lineages classified by Delfin et al. (2011)[21] were labelled as K2b1/K* for the purpose of Figure 1 (Main Text). It is likely however that the majority of these lineages fall into clade K2b1, as Karafet et al. (2015)[7] demonstrate with high resolution genotyping that K2b1 is present at a frequency of 0.60 in an Indigenous Philippine group. The results of this analysis hold rigorously if this dataset from Karafet et al. (2015)[7] is used, however the sample size (n=25) is significantly lower than that of Delfin et al. (2011)[21] (Frequencies: C - 0.089, D - 0, K2b1 - 0.32, NO - 0.6, Other - 0).

Haplogroup frequency estimates for a cosmopolitan Philippine population were obtained from Trejaut et al. (2014)[12]. This sample consisted of 146 Philippine Y-chromosome haplotypes from various populations of the Philippine archipelago (Frequencies: C - 0, D - 0, K2b1 - 0.069, NO - 0.85, Other - 0.082).

Malay hunter-gatherer and Malaysian – Data from Kutanan et al. (2019)[22] was summarised to provide Y-chromosome frequencies for Malay hunter-gatherer groups. These samples (n=4), which were all designated K* by Kutanan et al. (2019)[22] were labelled K2b1/K* for the purposes of Figure 2 (Main Text). These lineages are likely to belong to clade K2b1, as Karafet et al. (2015)[7] find 100% of Malay K(xNO/xR) lineages to belong to K2b1(Frequencies: C - 0, D - 0, K2b1 - 1, NO - 0, Other - 0).

For the Malaysian reference population the sample presented in Karafet et al. (2010)[5] was used. This dataset consists of 32 Malaysian individuals (Frequencies: C - 0.031, D - 0.031, K2b1 - 0.094, NO - 0.75, Other - 0.094).

v) Spatial Frequency Interpolation of Y-Chromosome Haplogroups

Below we provide a visual assessment of the concordance between observed Y-chromosome haplogroup frequencies and those interpolated using the kriging algorithm. We also present spatial frequency data for the remaining haplogroups mentioned in the Main Text, and separate plots for haplogroups N and O (Supplementary Figures 4-13).

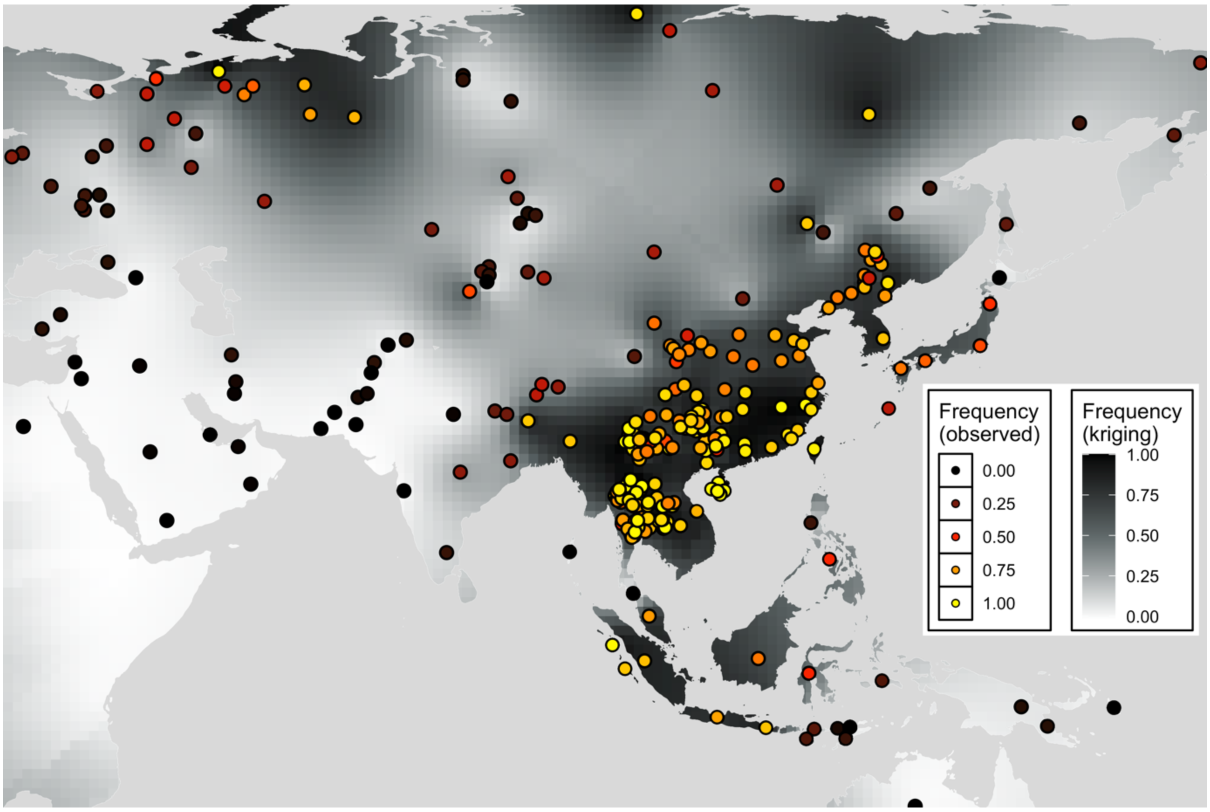

Supplementary Figure 4) Concordance between observed Y-chromosome haplogroup frequencies and those estimated using the kriging procedure for haplogroup NO. Darker surface shading indicates higher estimated haplotype frequency using kriging, and 'hotter' point colour (hottest denoted yellow) indicates higher observed haplotype frequency.

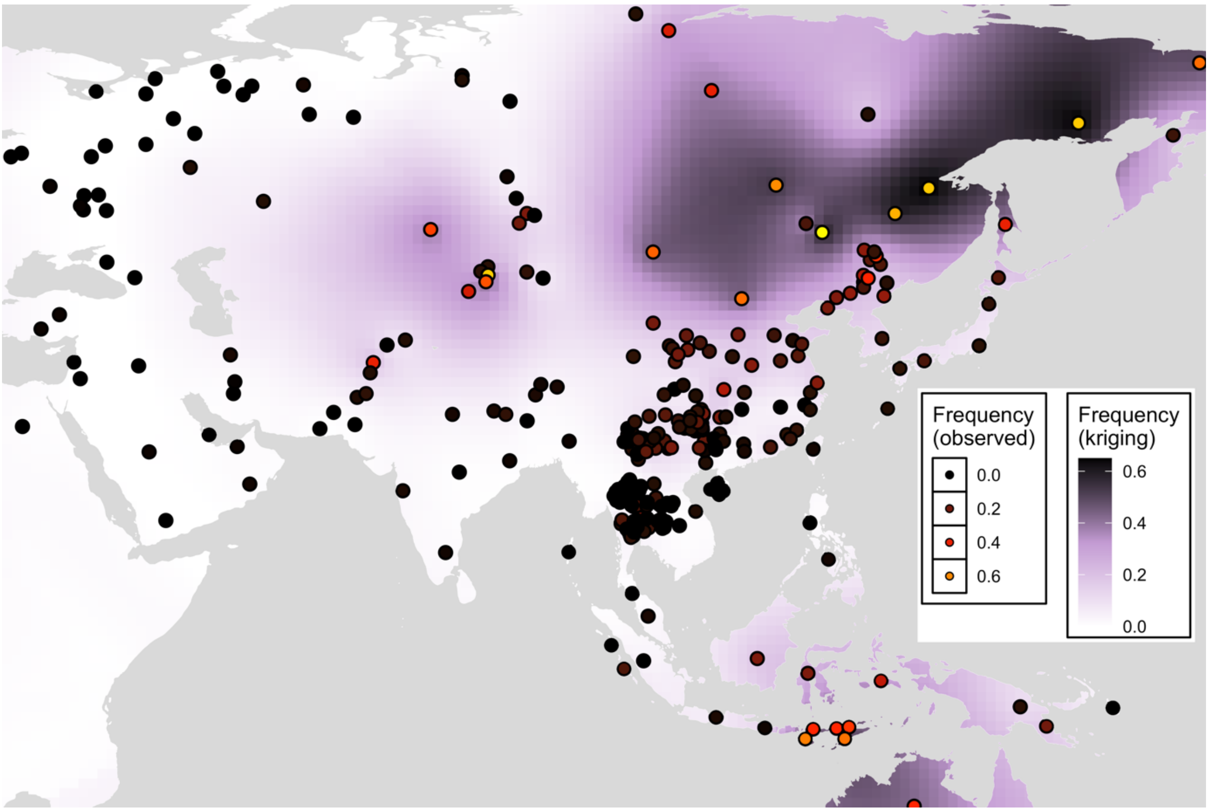

Supplementary Figure 5) Concordance between observed Y-chromosome haplogroup frequencies and those estimated using the kriging procedure for haplogroup C. Darker surface shading indicates higher estimated haplotype frequency using kriging, and ‘hotter’ point colour (hottest denoted yellow) indicates higher observed haplotype frequency.

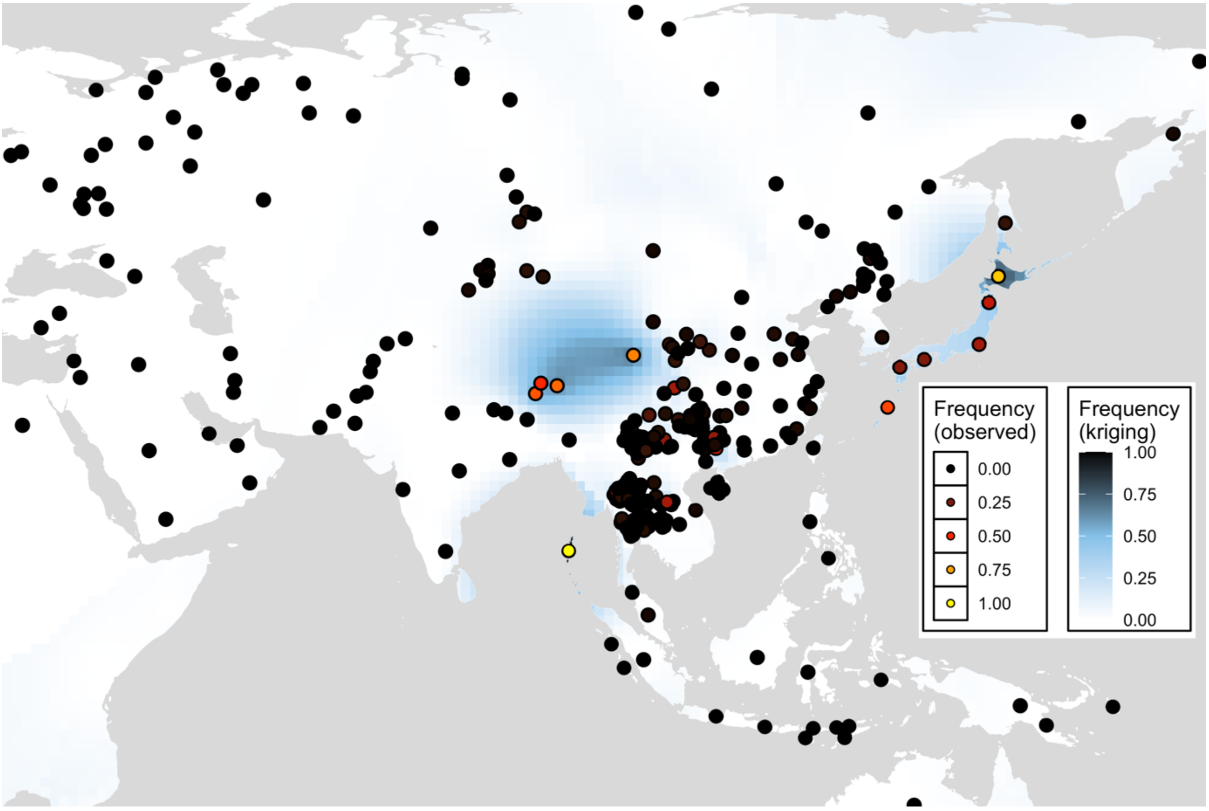

Supplementary Figure 6) Concordance between observed Y-chromosome haplogroup frequencies and those estimated using the kriging procedure for haplogroup D. Darker surface shading indicates higher estimated haplotype frequency using kriging, and 'hotter' point colour (hottest denoted yellow) indicates higher observed haplotype frequency.

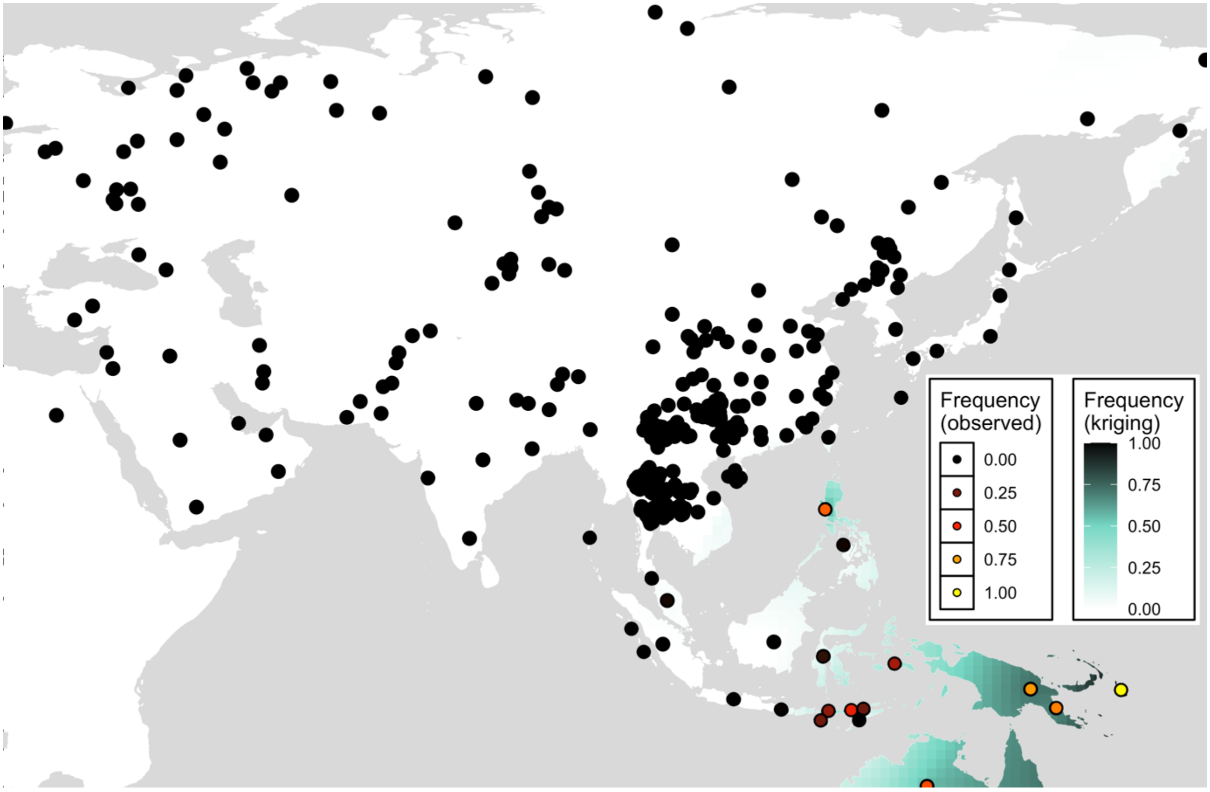

Supplementary Figure 7) Concordance between observed Y-chromosome haplogroup frequencies and those estimated using the kriging procedure for haplogroup K2b1. Darker surface shading indicates higher estimated haplotype frequency using kriging, and 'hotter' point colour (hottest denoted yellow) indicates higher observed haplotype frequency.

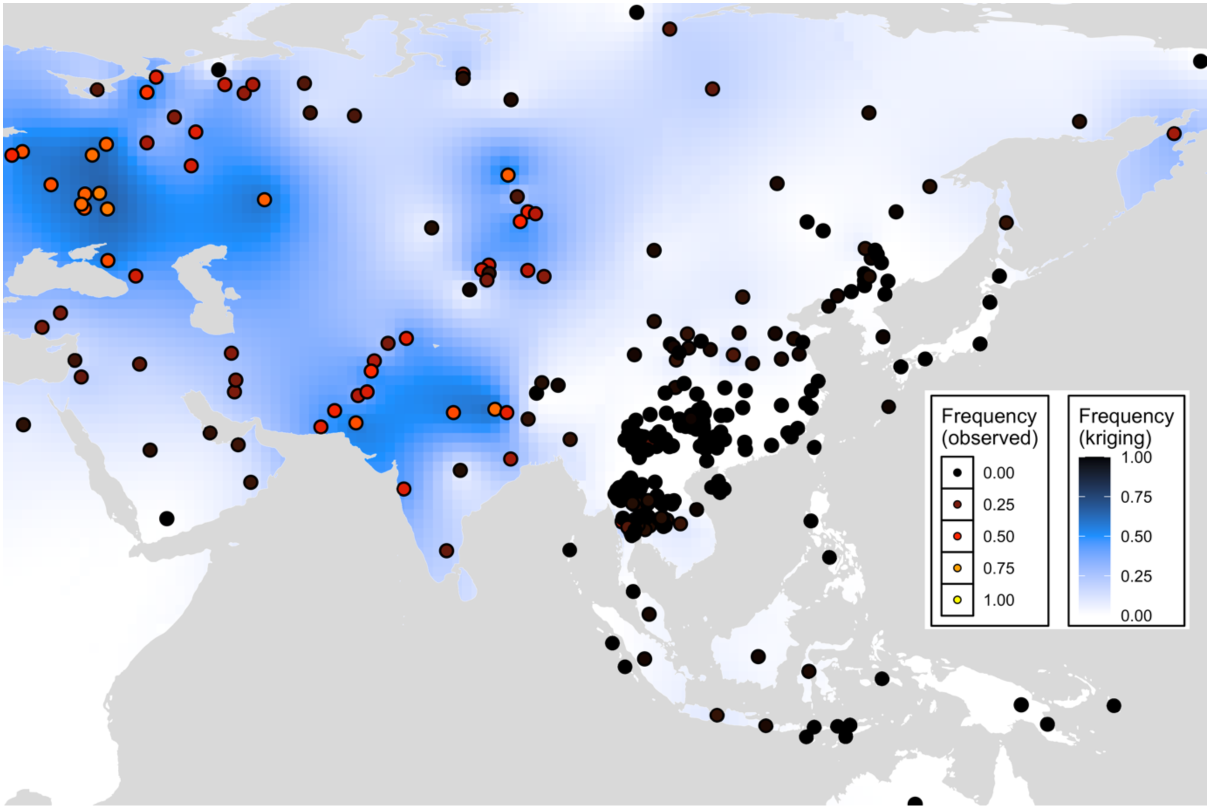

Supplementary Figure 8) Interpolation of Y-chromosome haplogroup R frequency. Darker surface shading indicates higher estimated haplotype frequency using kriging, and 'hotter' point colour (hottest denoted yellow) indicates higher observed haplotype frequency.

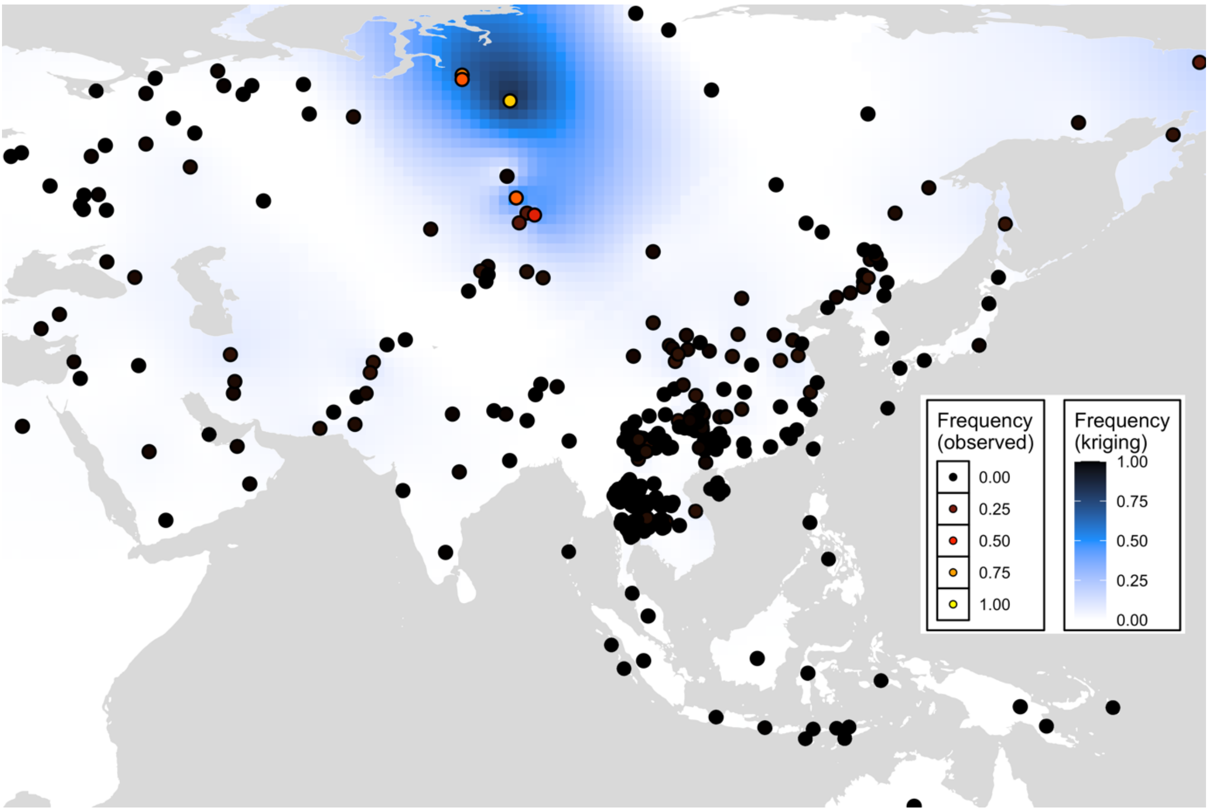

Supplementary Figure 9) Interpolation of Y-chromosome haplogroup Q frequency. Darker surface shading indicates higher estimated haplotype frequency using kriging, and 'hotter' point colour (hottest denoted yellow) indicates higher observed haplotype frequency.

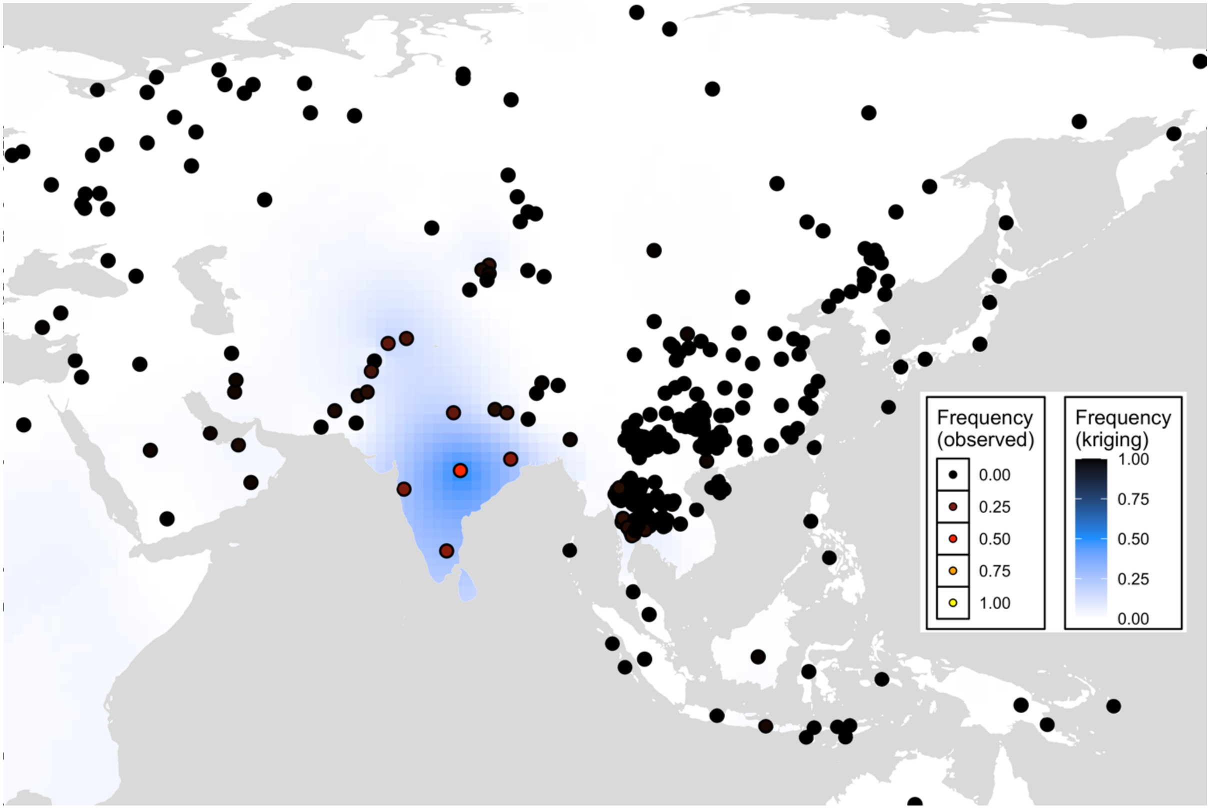

Supplementary Figure 10) Interpolation of Y-chromosome haplogroup H frequency. Darker surface shading indicates higher estimated haplotype frequency using kriging, and 'hotter' point colour (hottest denoted yellow) indicates higher observed haplotype frequency.

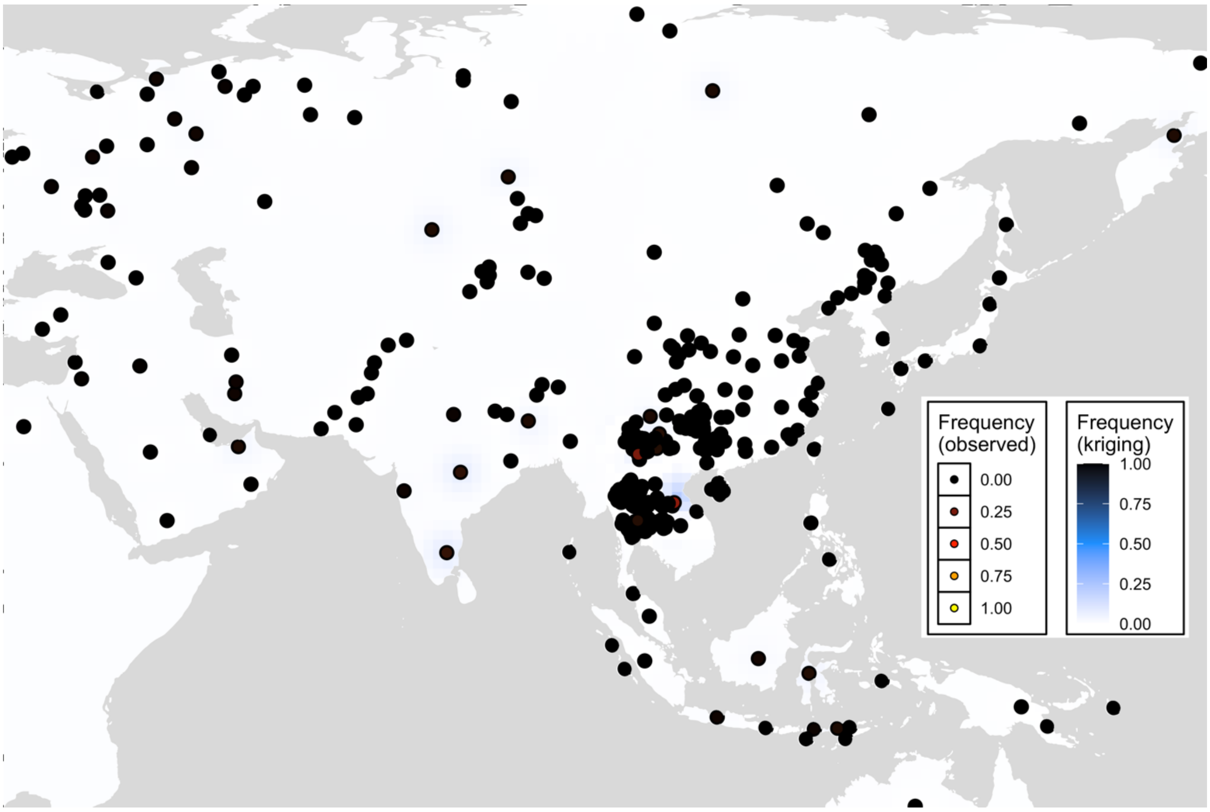

Supplementary Figure 11) Interpolation of Y-chromosome haplogroup F* frequency. Darker surface shading indicates higher estimated haplotype frequency using kriging, and 'hotter' point colour (hottest denoted yellow) indicates higher observed haplotype frequency.

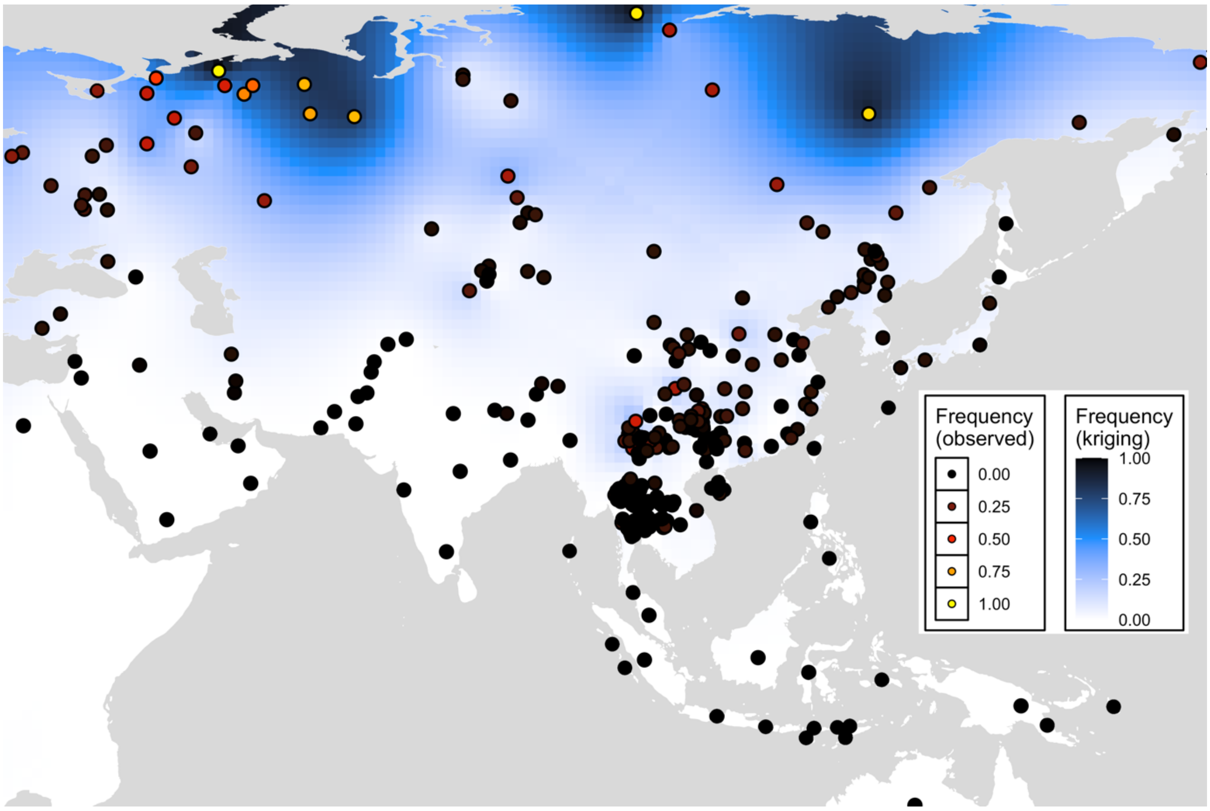

Supplementary Figure 12) Interpolation of Y-chromosome haplogroup N frequency. Darker surface shading indicates higher estimated haplotype frequency using kriging, and 'hotter' point colour (hottest denoted yellow) indicates higher observed haplotype frequency.

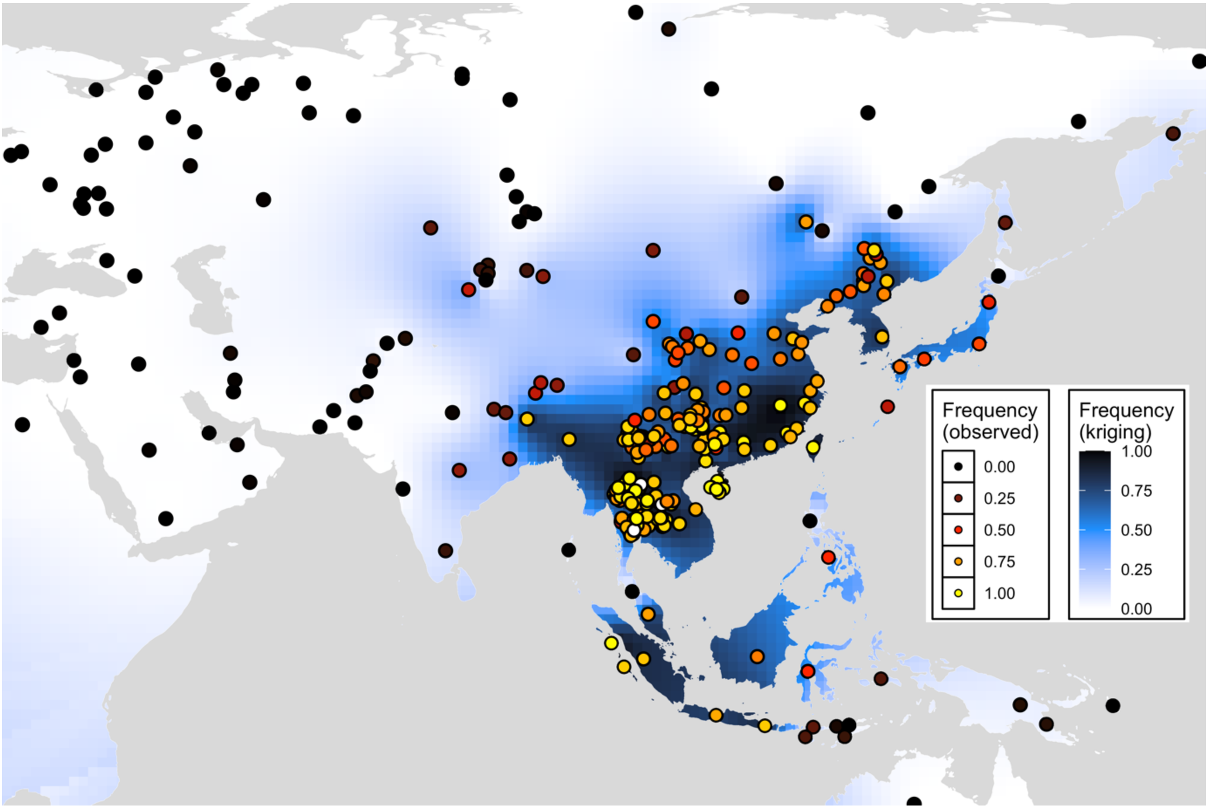

Supplementary Figure 13) Interpolation of Y-chromosome haplogroup O frequency. Darker surface shading indicates higher estimated haplotype frequency using kriging, and 'hotter' point colour (hottest denoted yellow) indicates higher observed haplotype frequency.

Supplementary Note 2. Phylogenetics

i) Bayesian Skyline Analysis

To explore the phylodynamics of key lineages, Bayesian Skyline plots were produced for each of the four key haplogroups analysed[23]. One of the key aspects which may vary in Bayesian Skyline analysis is the number of dimensions of the 'GroupSizes' and 'PopSizes' parameters, which determine the number of intervals over which population size is estimated[24]. Supplementary Figure 14 indicates that the shape of the trajectory and absolute magnitude of population size change for the four key lineages analysed remain largely consistent over multiple values of this parameter (varying from a minimum of 5 to the maximum value possible). Supplementary Figures 15 and 16 indicate that, when analysing lineages N and O separately, concurrent episodes of exponential population growth are observed for both lineages.

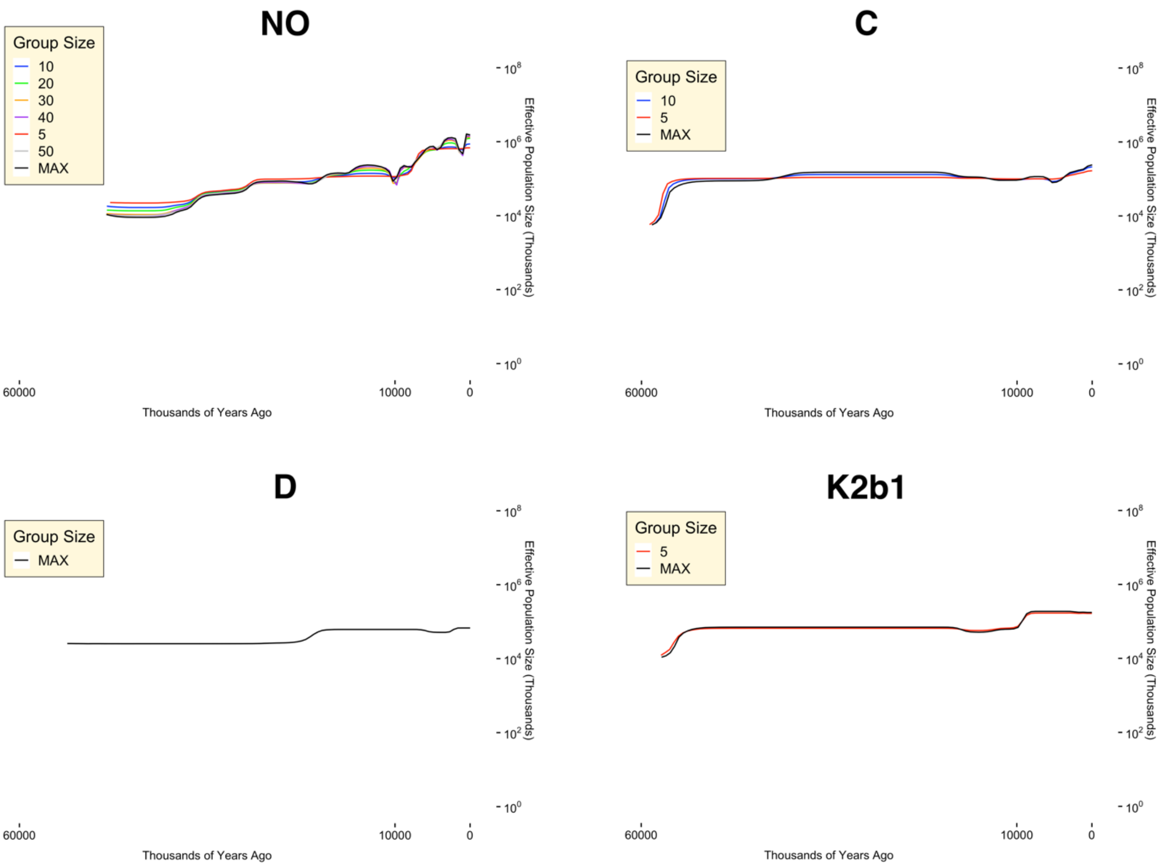

Supplementary Figure 14) Bayesian skyline traces for all key East Asian haplogroup lineages estimated using a different number of dimensions for the 'group size' parameter. Note that only one value appears for haplogroup D, due to the low number of sequences available for this haplogroup.

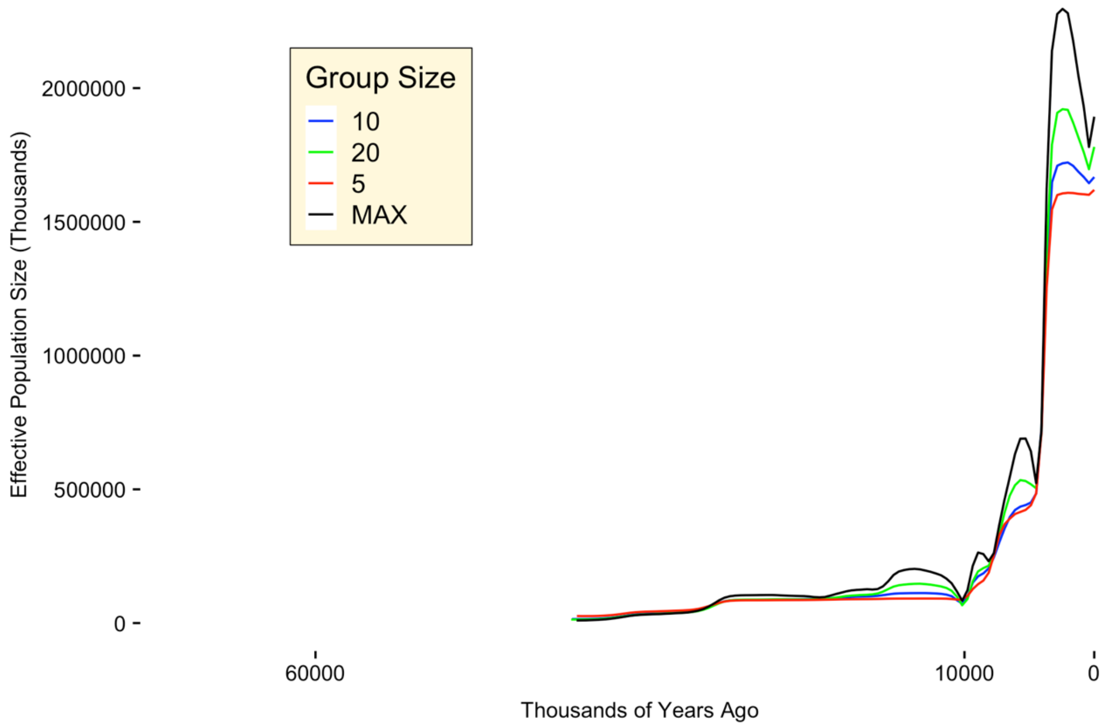

Supplementary Figure 15) Bayesian skyline traces for haplogroup O lineages only.

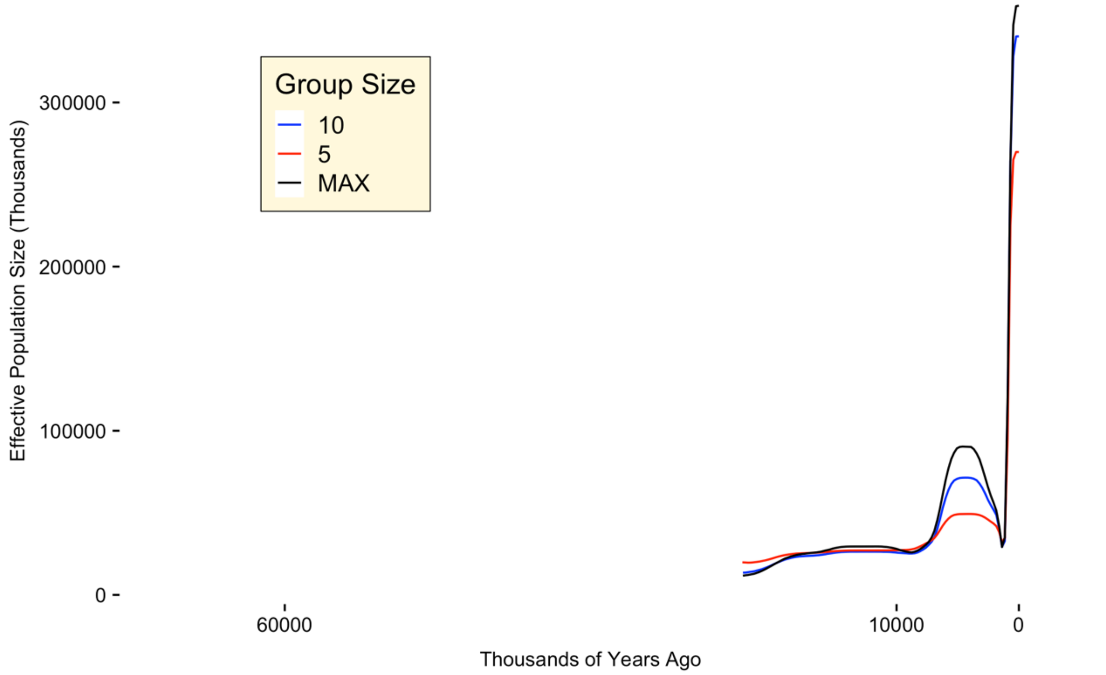

Supplementary Figure 16) Bayesian skyline traces for haplogroup N lineages only.

Supplementary Note 3. *Mtb* Lineage Haplogroup Frequency Analysis

i) Spatial Frequency Interpolation of *Mtb* Lineages

Included below are plots showing the concordance between observed *Mtb* lineage frequencies and those interpolated using kriging. Data for *Mtb* lineages 3 and 4 are also shown.

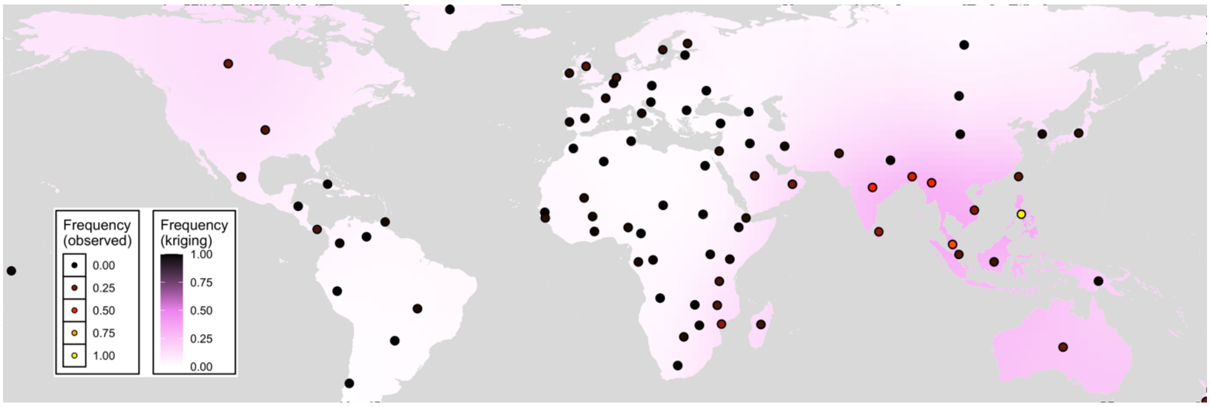

Supplementary Figure 17) Interpolation of *Mtb* lineage 1 frequency. Darker surface shading indicates higher estimated lineage frequency using kriging, and ‘hotter’ point colour (hottest denoted yellow) indicates higher observed lineage frequency.

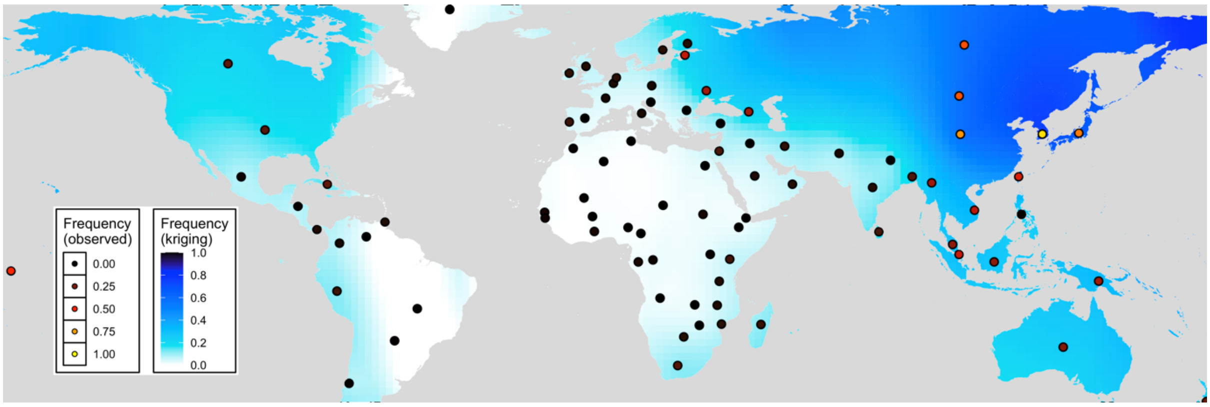

Supplementary Figure 18) Interpolation of *Mtb* lineage 2 frequency. Darker surface shading indicates higher estimated lineage frequency using kriging, and ‘hotter’ point colour (hottest denoted yellow) indicates higher observed lineage frequency.

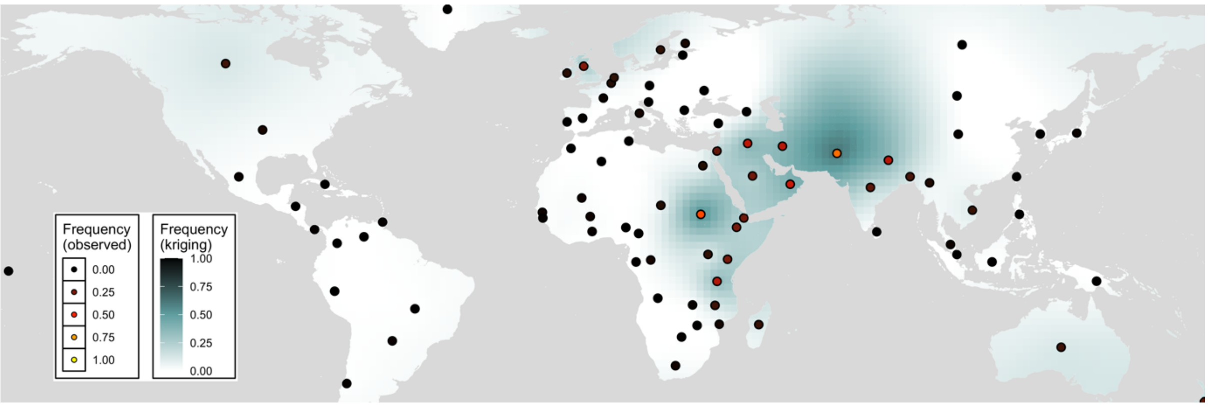

Supplementary Figure 19) Interpolation of *Mtb* lineage 3 frequency. Darker surface shading indicates higher estimated lineage frequency using kriging, and 'hotter' point colour (hottest denoted yellow) indicates higher observed lineage frequency.

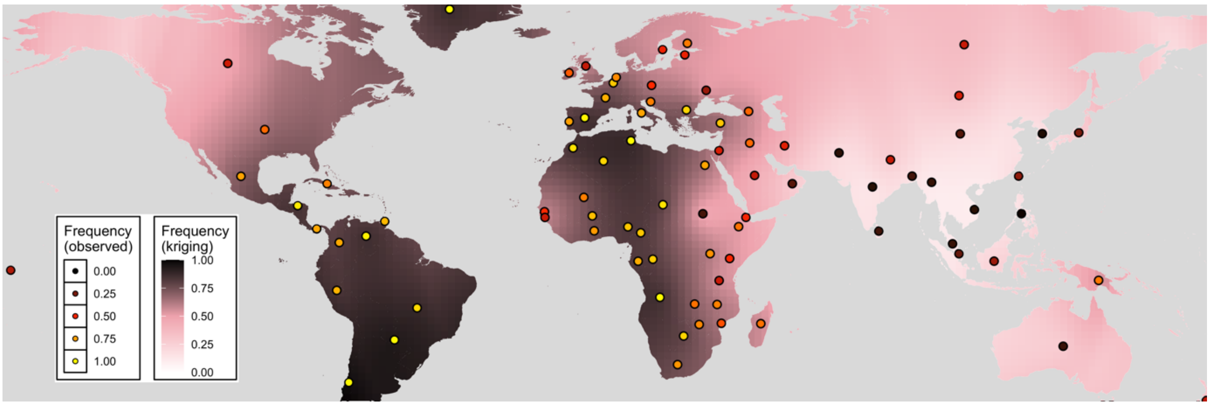

Supplementary Figure 20) Interpolation of *Mtb* lineage 4 frequency. Darker surface shading indicates higher estimated lineage frequency using kriging, and 'hotter' point colour (hottest denoted yellow) indicates higher observed lineage frequency.

ii) Correlation Between Putative First and Second Layer *Mtb* and Y-Chromosome lineages

We compared the frequencies of putative first and second layer *Mtb* and Y-chromosome lineages across countries or major geographic regions. To obtain these frequencies for the Y-chromosome, we partitioned all populations considered in the kriging dataset (Supplementary Figure 1) into their respective country, before taking the mean frequency of each haplogroup across all populations within that country. *Mtb* lineage frequencies per country were obtained from the analysis of Wiens et al. (2018)[25].

To account for the fact that some countries cover a broad geographical area and may display heterogenous frequencies of Y-chromosome or *Mtb* lineages, we subdivided large countries into major geographical regions. Indonesia, which displays a strong gradient in Y-chromosome haplogroup frequencies[14], we split into sampling locations from the islands of Borneo, Timor, Java and Sulewasi, and we also considered a subset of the Western Russian populations from our survey. *Mtb* lineage frequencies from each of these regions were obtained from various studies (Borneo[26]; Timor and Java[27]; Sulewasi[28]; West Russia[29]). Correlations between *Mtb* and Y-chromosome haplogroup frequencies were calculated using the cor.test() function of the 'stats' package of R[30].

When assessing the correlation in frequencies between putative first layer Y-chromosome haplogroups and *Mtb* lineage 1, we excluded the country of India from consideration. As elaborated upon in the Discussion, we believe the presence of L1 in South Asia is linked to the arrival of its initial inhabitants, the Ancestral Southern Indians, who share genomic affinities with the Andamanese, and by extension, the other first layer populations of East Asia[31] (see Discussion). We saw the incorporation of these groups as being outside the scope of the model we propose, as the Two Layer hypothesis typically only describes the peopling of East Asia. We note that the correlation between putative first layer *Mtb* and Y-

chromosome lineage frequencies increases when modelling haplogroup H as a first layer haplogroup and including India in the calculation (Spearman's rho = 0.45; p=0.032).

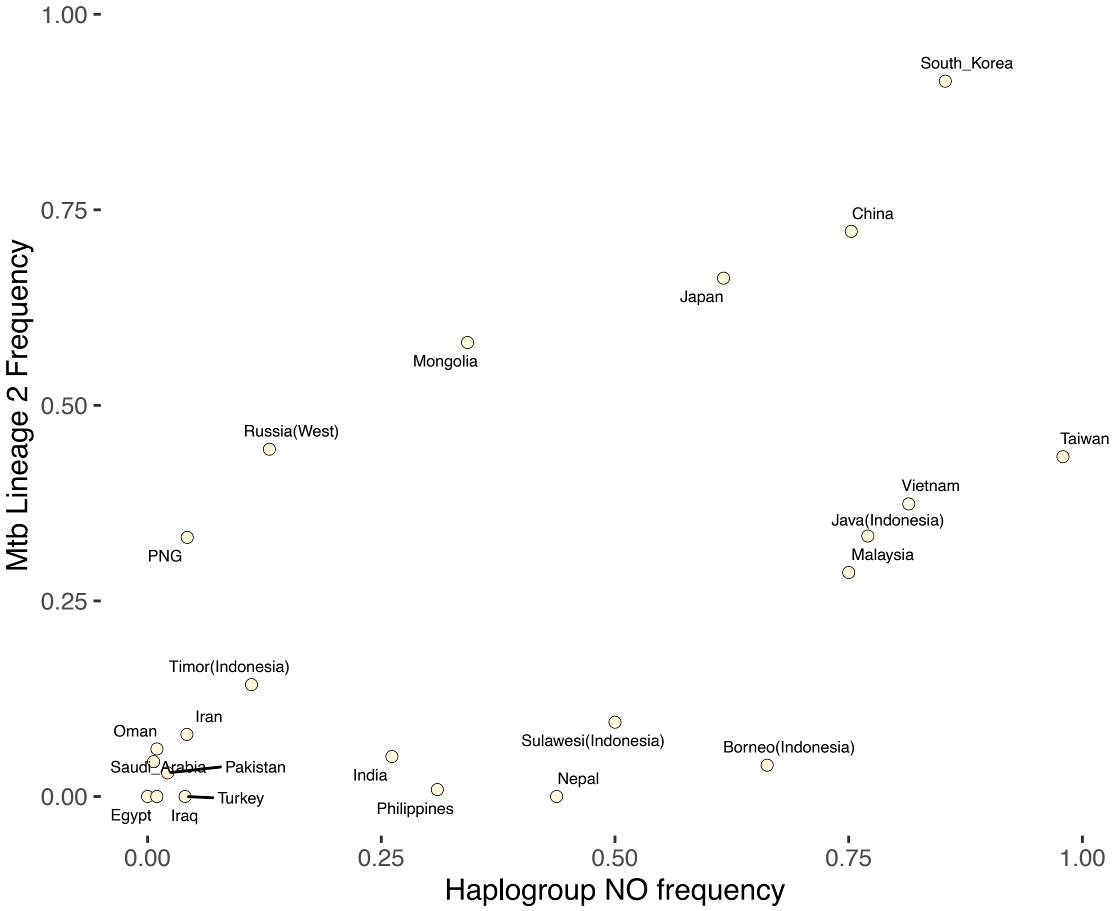

Supplementary Figure 21) Scatterplot showing correlation in the frequency of haplogroup NO and *Mtb* lineage 2 across countries or major geographical regions.

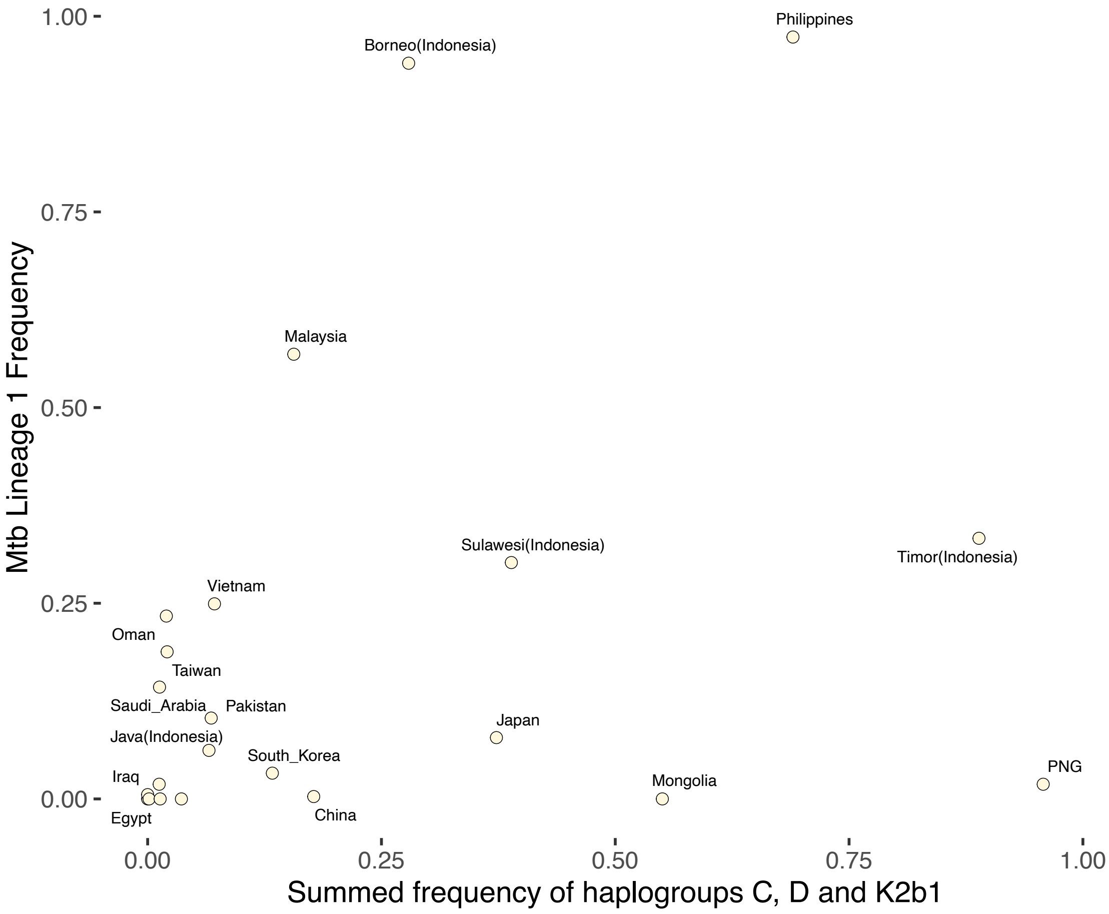

Supplementary Figure 22) Scatterplot showing correlation in the summed frequencies of haplogroups C, D and K2b1/K* and *Mtb* lineage 1 across countries or major geographical regions.

Supplementary Note 4. Comparing the dynamics of the *Mtb* and Y-chromosome phylogenies

We conducted a quantitative comparison of lineage through time (LTT) trajectories for the *Mtb* lineages and Y-chromosome haplogroups we propose to be associated with the first and second layers of peopling. To do this, we interpolated the mean LTT count for all haplogroups and lineages at regular intervals across the LTT trajectories output by TRACER (based on the phylogenies inferred using BEAST). We then calculated the absolute rate of lineage increase across these intervals by subtracting the count at the previous interval from the present interval (Supplementary Figure 23).

We compared the magnitudes of these values using one-sided paired Wilcoxon tests for the intervals over which the trajectories of each pair of lineages overlap (to account for demographic influences likely to affect both lineages, like the Neolithic expansion). These tests were carried out using the 'stats' package of R (R Core Team, 2021)[30]. The comparisons

conducted included *Mtb* lineages 1 vs 2, and Y-chromosome haplogroups NO vs C, NO vs D and NO vs K2b1. In all comparisons, the growth rate of putative second layer lineages was significantly higher than that of putative first layer lineages (NO vs C, $p<2.2\times10^{-16}$; NO vs D, $p<2.2\times10^{-16}$; NO vs K2b1, $p<2.2\times10^{-16}$ and L2 vs L1, $p=0.038$). Similar results were returned when considering growth rates from the Neolithic period (10Kya) until the present (NO vs C, $p=2.98\times10^{-8}$; NO vs D, $p=2.98\times10^{-8}$; NO vs K2b1, $p=2.98\times10^{-8}$ and L2 vs L1, $p=2.62\times10^{-6}$).

We also calculated a relative rate of increase for each lineage by dividing the lineage count in each interval by the count in the previous interval. Again, second layer lineages displayed higher growth rates during the period where trajectories overlapped (NO vs C, $p<6.13\times10^{-10}$; NO vs D, $p=9.55\times10^{-11}$; NO vs K2b1, $p=5.70\times10^{-14}$ and L2 vs L1, $p=5.91\times10^{-7}$). During the Neolithic period, all comparisons were significant (NO vs D, $p=6.65\times10^{-3}$; NO vs K2b1, $p=3.61\times10^{-3}$ and L2 vs L1, $p=5.19\times10^{-5}$), aside from NO vs C ($p=0.41$).

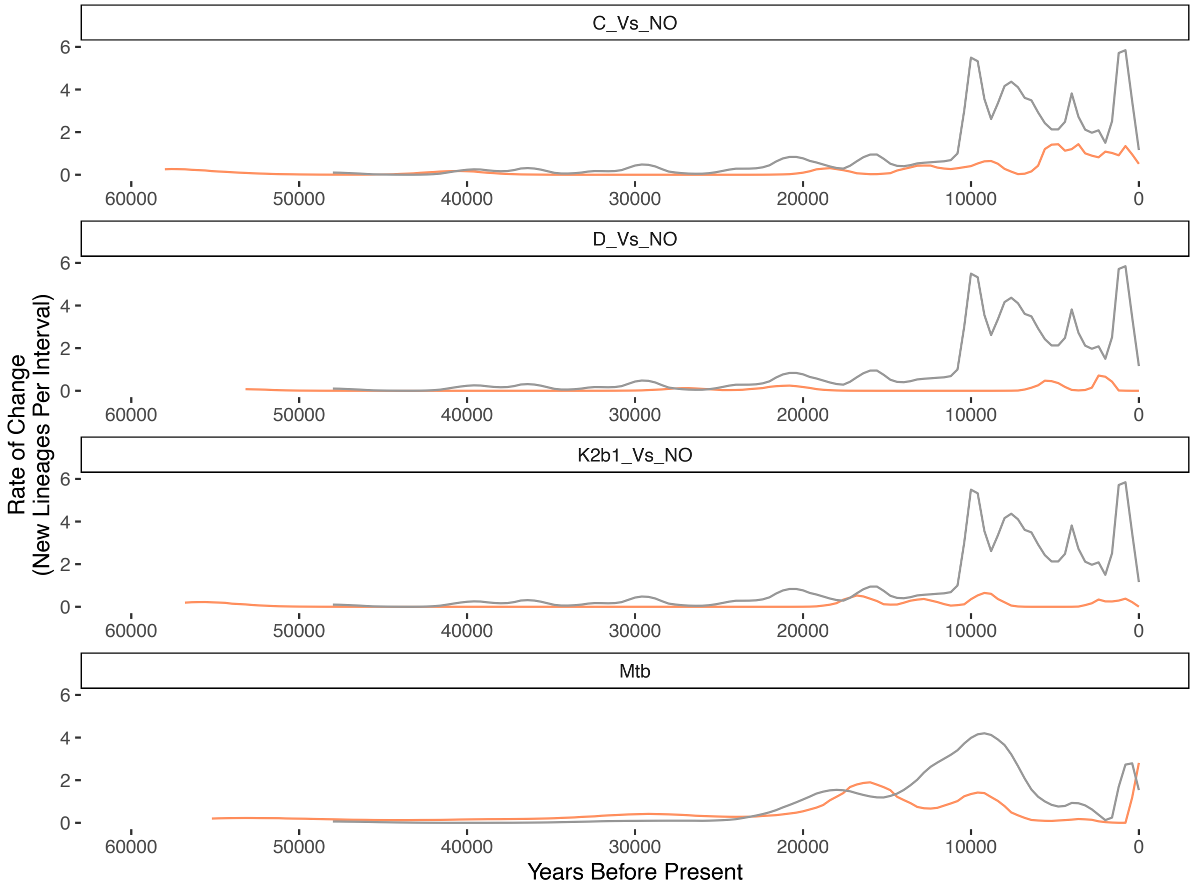

Supplementary Figure 23) Absolute growth rates of each *Mtb* and Y-chromosome lineage or haplogroup, based on the LTT trajectories generated using TRACER. Rates were calculated at regular intervals from timepoint zero to the coalescence of that lineage or haplogroup. Rates for putative first layer lineages are coloured in orange, and rates for putative second layer lineages in gray.

Supplementary Note 5. Comparing *Mtb* node ages under alternate dating scenarios

We compiled a table listing the *Mtb* node ages and substitution rates inferred by several prior studies[32,33,34,35,36]. These ages and rates were obtained from either the main text or supplementary material of each respective study.

For the two models proposed by Comas et al. (2013)[36], we retrieved the node ages from the phylogenies presented in their Supplementary Material. We obtained the substitution rate for the MTBC-70 model from the main text of their article, and derived the MTBC-185 substitution rate by converting the rate in their table (Table 1 in Comas et al. (2013)[36]) into a genome wide rate per year (as opposed to a rate across polymorphic sites per 1,000 years). Note that in Table 1 from Comas et al. (2013)[36], the phrase "coalescent time for Lineage 1" refers to the date lineage 1 coalesces with modern *Mtb* lineages (i.e. what we refer to as "L1 & L2 coalescence age" in Table S1 below).

Study/ model	L1 node age (Kya)	L2 node age (Kya)	L1 & L2 coalescence age (Kya)	Substitution rate (subs. per site per year)
Menardo et al. (2021) * [32]	~ 0.85	Not included	Not included	1.4×10^{-7}
O'Neill et al. (2019) **[34]	2.38	2.04	4.70	5.0×10^{-8}
Comas et al. (2013) [36] MTBC–70	38.0	32.0	66.9	2.58×10^{-9}
This study	56.5	48.9	112.4	2.16×10^{-9}
Comas et al. (2013) [36] MTBC–185	103.0	87.8	183.0	9.42×10^{-10}

Supplementary Table 1) Node ages of key *Mtb* lineages inferred in several studies, along with the corresponding substitution rate used or inferred by each. Note that Menardo et al. (2021)[32] did not report the age of the L2 node or the L1 & L2 coalescence age, as their study focused on lineages 1 and 3.
*Menardo et al. (2021)[32] use a substitution rate inferred by Menardo et al. (2019)[33], which was obtained from tip dating of contemporary samples (all less than 40 years old).
**O'Neill et al. (2019)[34] used the substitution rate inferred by Kay et al. (2015)[35], which was based on tip calibration, incorporating samples collected during the 18th century AD.

Supplementary References

1. Zhong, H. *et al.* Extended Y Chromosome Investigation Suggests Postglacial Migrations of Modern Humans into East Asia via the Northern Route. *Molecular Biology and Evolution*, **28**, 717–727 (2011).

2. Abu-Amero, K. K. *et al.* Saudi Arabian Y-Chromosome diversity and its relationship with nearby regions. *BMC Genetics*, **10**, 59; 10.1186/1471-2156-10-59 (2009).

3. Kutanan, W. *et al.* Contrasting Paternal and Maternal Genetic Histories of Thai and Lao Populations. *Molecular Biology and Evolution*, **36**, 1490–1506 (2019).

4. Tambets, K. *et al.* Genes reveal traces of common recent demographic history for most of the Uralic-speaking populations. *Genome Biology*, **19**, 139; 10.1186/s13059-018-1522-1 (2018).

5. Karafet, T. M. *et al.* Major East-West Division Underlies Y Chromosome Stratification across Indonesia. *Molecular Biology and Evolution*, **27**, 1833–1844 (2010).

6. Karafet, T. M. *et al.* New binary polymorphisms reshape and increase resolution of the human Y chromosomal haplogroup tree. *Genome Research*, **18**, 830–838 (2008).

7. Karafet, T. M., Mendez, F. L., Sudoyo, H., Lansing, J. S., & Hammer, M. F. Improved phylogenetic resolution and rapid diversification of Y-chromosome haplogroup K-M526 in Southeast Asia. *European Journal of Human Genetics*, **23**, 369–373 (2015).

8. Trivedi, R. *et al.* Genetic Imprints of Pleistocene Origin of Indian Populations: A Comprehensive Phylogeographic Sketch of Indian Y-Chromosomes. *International Journal of Human Genetics*, **8**, 97–118 (2008).

9. Cordaux, R. *et al.* Independent Origins of Indian Caste and Tribal Paternal Lineages. *Current Biology*, **14**, 231–235 (2004).

10. Peng, M. S. *et al.* Retrieving Y chromosomal haplogroup trees using GWAS data. *European Journal of Human Genetics*, **22**, 1046–1050 (2014).

11. Nonaka, I., Minaguchi, K., & Takezaki, N. Y-chromosomal Binary Haplogroups in the Japanese Population and their Relationship to 16 Y-STR Polymorphisms. *Annals of Human Genetics*, **71**, 480–495 (2007).

12. Trejaut, J. A. *et al.* Taiwan Y-chromosomal DNA variation and its relationship with Island Southeast Asia. *BMC Genetics*, **15**, 77; 10.1186/1471-2156-15-77 (2014).

13. Thangaraj, K. *et al.* Genetic Affinities of the Andaman Islanders, a Vanishing Human Population. *Current Biology*, **13**, 86–93 (2003).

14. Kayser, M. *et al.* Melanesian and Asian Origins of Polynesians: mtDNA and Y Chromosome Gradients Across the Pacific. *Molecular Biology and Evolution*, **23**, 2234–2244 (2006).

15. Bergström, A. *et al.* A Neolithic expansion, but strong genetic structure, in the independent history of New Guinea. *Science*, **357**, 1160–1163 (2017).

16. Mirabal, S., Cadenas, A. M., Garcia-Bertrand, R., & Herrera, R. J. Ascertaining the role of Taiwan as a source for the Austronesian expansion. *American Journal of Physical Anthropology*, **150**, 551–564 (2013).

17. Lipson, M. *et al.* Reconstructing Austronesian population history in Island Southeast Asia. *Nature Communications*, **5**, 4689; 10.1038/ncomms5689 (2014).

18. Ko, A. M. S. *et al.* Early Austronesians: Into and Out Of Taiwan. *The American Journal of Human Genetics*, **94**, 426–436 (2014).

19. Tajima, A. *et al.* Genetic origins of the Ainu inferred from combined DNA analyses of maternal and paternal lineages. *Journal of Human Genetics*, **49**, 187–193 (2004).

20. Hammer, M. F. *et al.* Dual origins of the Japanese: common ground for hunter-gatherer and farmer Y chromosomes. *Journal of Human Genetics*, **51**, 47–58 (2005).

21. Delfin, F. *et al.* The Y-chromosome landscape of the Philippines: extensive heterogeneity and varying genetic affinities of Negrito and non-Negrito groups. *European Journal of Human Genetics*, **19**, 224–230 (2011).

22. Kutanan, W. *et al.* Contrasting Paternal and Maternal Genetic Histories of Thai and Lao Populations. *Molecular Biology and Evolution*, **36**, 1490–1506 (2019).

23. Drummond, A. J. Bayesian Coalescent Inference of Past Population Dynamics from Molecular Sequences. *Molecular Biology and Evolution*, **22**, 1185–1192 (2005).

24. Ho, S. Y. W., & Shapiro, B. Skyline-plot methods for estimating demographic history from nucleotide sequences. *Molecular Ecology Resources*, **11**, 423–434 (2011).

25. Wiens, K. E. *et al.* Global variation in bacterial strains that cause tuberculosis disease: a systematic review and meta-analysis. *BMC Medicine*, **16**, 196; 10.1186/s12916-018-1180-x (2018).

26. Bainomugisa, A. *et al.* Genomic epidemiology of tuberculosis in eastern Malaysia: insights for strengthening public health responses. *Microbial Genomics*, **7**, 000573; 10.1099/mgen.0.000573 (2021).

27. Parwati, I. *et al.* Mycobacterium tuberculosis Population Structures Differ Significantly on Two Indonesian Islands. *Journal of Clinical Microbiology*, **46**, 3639–3645 (2008).

28. Sasmono, R. T. *et al.* Heterogeneity of Mycobacterium tuberculosis strains in Makassar, Indonesia. *The International Journal of Tuberculosis and Lung Disease*, **16**, 1441–1448 (2012).

29. Mokrousov, I. *et al.* Mycobacterium tuberculosis Population in Northwestern Russia: An Update from Russian-EU/Latvian Border Region. *PLos ONE*, **7**, e41318; 10.1371/journal.pone.0041318 (2012).

30. R Core Team R: A language and environment for statistical computing. R Foundation for Statistical Computing, Vienna, Austria. https://www.R-project.org/ (2021).

31. Reich, D., Thangaraj, K., Patterson, N., Price, A. L., & Singh, L. Reconstructing Indian population history. *Nature*, **461**, 489–494 (2009).

32. Menardo, F. *et al.* Local adaptation in populations of Mycobacterium tuberculosis endemic to the Indian Ocean Rim. *F1000Research*, **10**, 60; 10.12688/f1000research.28318.2 (2021).

33. Menardo, F., Duchêne, S., Brites, D., & Gagneux, S. The molecular clock of Mycobacterium tuberculosis. *PLOS Pathogens*, **15**, e1008067; 10.1371/journal.ppat.1008067 (2019).

34. O'Neill, M. B. *et al.* Lineage specific histories of Mycobacterium tuberculosis dispersal in Africa and Eurasia. *Molecular Ecology*, **28**, 3241–3256 (2019).

35. Kay, G. L. *et al.* Eighteenth-century genomes show that mixed infections were common at time of peak tuberculosis in Europe. *Nature Communications*, **6**, 6717; 10.1038/ncomms7717 (2015).

36. Comas, I. *et al.* Out-of-Africa migration and Neolithic coexpansion of Mycobacterium tuberculosis with modern humans. *Nature Genetics*, **45**, 1176–1182 (2013).
