# Supplementary material for: Parallel signatures of Mycobacterium tuberculosis and human Y-chromosome phylogeography support the Two Layer model of East Asian population history
Source: Commun Biol. 2023 Oct 13;6:1037. doi: 10.1038/s42003-023-05388-8 (PMC10575886; doi:10.1038/s42003-023-05388-8)
Supplement: Supplementary file 3 — Description of Additional Supplementary Files [file 42003_2023_5388_MOESM3_ESM.docx]

**Description of Additional Supplementary Files**

**File name:** Supplementary Data 1

**Description:** The raw data used to plot figures 2c and 5b-c.
