# Supplementary material for: Parallel signatures of Mycobacterium tuberculosis and human Y-chromosome phylogeography support the Two Layer model of East Asian population history
Source: Commun Biol. 2023 Oct 13;6:1037. doi: 10.1038/s42003-023-05388-8 (PMC10575886; doi:10.1038/s42003-023-05388-8)
Supplement: Supplementary file 5 — Reporting Summary [file 42003_2023_5388_MOESM5_ESM.pdf]

nature portfolio

Corresponding author(s): Dr. Matthew Silcocks

Last updated by author(s): 18th July 2023

Reporting Summary

Nature Portfolio wishes to improve the reproducibility of the work that we publish. This form provides structure for consistency and transparency in reporting. For further information on Nature Portfolio policies, see our Editorial Policies and the Editorial Policy Checklist.

Please do not complete any field with "not applicable" or n/a. Refer to the help text for what text to use if an item is not relevant to your study.
For final submission: please carefully check your responses for accuracy; you will not be able to make changes later.

Statistics

For all statistical analyses, confirm that the following items are present in the figure legend, table legend, main text, or Methods section.

n/a	Confirmed	
☐	☑	The exact sample size (n) for each experimental group/condition, given as a discrete number and unit of measurement
☒	☐	A statement on whether measurements were taken from distinct samples or whether the same sample was measured repeatedly
☐	☑	The statistical test(s) used AND whether they are one- or two-sided<br>*Only common tests should be described solely by name; describe more complex techniques in the Methods section.*
☒	☐	A description of all covariates tested
☐	☑	A description of any assumptions or corrections, such as tests of normality and adjustment for multiple comparisons
☐	☑	A full description of the statistical parameters including central tendency (e.g. means) or other basic estimates (e.g. regression coefficient) AND variation (e.g. standard deviation) or associated estimates of uncertainty (e.g. confidence intervals)
☐	☑	For null hypothesis testing, the test statistic (e.g. F, t, r) with confidence intervals, effect sizes, degrees of freedom and P value noted<br>*Give P values as exact values whenever suitable.*
☐	☑	For Bayesian analysis, information on the choice of priors and Markov chain Monte Carlo settings
☒	☐	For hierarchical and complex designs, identification of the appropriate level for tests and full reporting of outcomes
☐	☑	Estimates of effect sizes (e.g. Cohen's d, Pearson's r), indicating how they were calculated

Our web collection on statistics for biologists contains articles on many of the points above.

Software and code

Policy information about availability of computer code

Data collection	No novel algorithms, software or protocols were used for the collection of this data.
Data analysis	No novel algorithms, software or protocols were used for the analysis of this data. All existing software packages used are referenced in the text.

For manuscripts utilizing custom algorithms or software that are central to the research but not yet described in published literature, software must be made available to editors and reviewers. We strongly encourage code deposition in a community repository (e.g. GitHub). See the Nature Portfolio guidelines for submitting code & software for further information.

Data

Policy information about availability of data

All manuscripts must include a data availability statement. This statement should provide the following information, where applicable:

- Accession codes, unique identifiers, or web links for publicly available datasets
- A description of any restrictions on data availability
- For clinical datasets or third party data, please ensure that the statement adheres to our policy

This analysis used only previously published data, available from the ncbi sequence read archive (https://www.ncbi.nlm.nih.gov/sra), or presented in the main text or supplementary material of prior studies

Research involving human participants, their data, or biological material

Policy information about studies with human participants or human data. See also policy information about sex, gender (identity/presentation), and sexual orientation and race, ethnicity and racism.

Reporting on sex and gender	NA
Reporting on race, ethnicity, or other socially relevant groupings	NA
Population characteristics	Data was only drawn from studies with no evidence of biased ascertainment in their sampling regime (i.e. preferentially selecting Y-chromosomes known to belong to a particular haplogroup).
Recruitment	See above. Additionally, we only used Mtb sequences from studies which did not preferentially recruit patients with drug resistant strains.
Ethics oversight	NA

Note that full information on the approval of the study protocol must also be provided in the manuscript.

Field-specific reporting

Please select the one below that is the best fit for your research. If you are not sure, read the appropriate sections before making your selection.

☑ Life sciences ☐ Behavioural & social sciences ☐ Ecological, evolutionary & environmental sciences

For a reference copy of the document with all sections, see nature.com/documents/nr-reporting-summary-flat.pdf

Life sciences study design

All studies must disclose on these points even when the disclosure is negative.

Sample size	As this analysis relied on publicly available data, sample sizes were dictated by availability. Mtb sequences used for the phylogeny were randomly down-sampled to match the number of Y-chromosome sequences (as specified in the Methods), to limit biases in the subsequent phylogenetic inferences.
Data exclusions	Exclusion of samples was carried out only on the basis of sequence data quality, as specified in the Methods.
Replication	Multiple independent replicates of the BEAST phylogenetic analysis were run to check for consistency, and skyline trajectories were replicated after running BEAST with different parameter values (see Supplementary Notes and Figures).
Randomization	Aside from the random down-sampling of Mtb sequences mentioned above, this study did not involve analyses in which randomisation was necessary.
Blinding	The authors did not carry out blinding in this analysis.

Reporting for specific materials, systems and methods

We require information from authors about some types of materials, experimental systems and methods used in many studies. Here, indicate whether each material, system or method listed is relevant to your study. If you are not sure if a list item applies to your research, read the appropriate section before selecting a response.

Materials & experimental systems

n/a	Involved in the study	
X	☐	Antibodies
X	☐	Eukaryotic cell lines
X	☐	Palaeontology and archaeology
X	☐	Animals and other organisms
X	☐	Clinical data
X	☐	Dual use research of concern
X	☐	Plants

Methods

n/a	Involved in the study	
X	☐	ChIP-seq
X	☐	Flow cytometry
X	☐	MRI-based neuroimaging
