# Supplementary material for: Parallel signatures of Mycobacterium tuberculosis and human Y-chromosome phylogeography support the Two Layer model of East Asian population history
Source: Commun Biol. 2023 Oct 13;6:1037. doi: 10.1038/s42003-023-05388-8 (PMC10575886; doi:10.1038/s42003-023-05388-8)
Supplement: Supplementary file 1 — Peer Review File [file 42003_2023_5388_MOESM1_ESM.pdf]

nature portfolio

Peer Review File

Parallel signatures of Mycobacterium tuberculosis and human Y-chromosome phylogeography support the Two Layer model of East Asian population history

Open Access This file is licensed under a Creative Commons Attribution 4.0 International License, which permits use, sharing, adaptation, distribution and reproduction in any medium or format, as long as you give appropriate credit to the original author(s) and the source, provide a link to the Creative Commons license, and indicate if changes were made. In the cases where the authors are anonymous, such as is the case for the reports of anonymous peer reviewers, author attribution should be to 'Anonymous Referee' followed by a clear attribution to the source work. The images or other third party material in this file are included in the article's Creative Commons license, unless indicated otherwise in a credit line to the material. If material is not included in the article's Creative Commons license and your intended use is not permitted by statutory regulation or exceeds the permitted use, you will need to obtain permission directly from the copyright holder. To view a copy of this license, visit http://creativecommons.org/licenses/by/4.0/.

This manuscript has been previously reviewed at another Nature Portfolio journal. This document only contains reviewer comments and rebuttal letters for versions considered at Communications Biology.

REVIEWERS' COMMENTS:

Reviewer #3 (Remarks to the Author):

The authors present a revised version of their article, in which they substantially extended their discussion by elaborating on their approach of node dating vs. tip dating when calibrating the time tree for M. tuberculosis. In addition, a comprehensive comparison with the results of models from other publications is now provided.

Furthermore, the authors have performed additional analyses in order to support their model.

All my comments were sufficiently addressed. I have no further suggestions.
